# Composition of the Biofilm Matrix of *Cutibacterium acnes* Acneic Strain RT5

**DOI:** 10.3389/fmicb.2019.01284

**Published:** 2019-06-21

**Authors:** Andrei V. Gannesen, Evelina L. Zdorovenko, Ekaterina A. Botchkova, Julie Hardouin, Sebastien Massier, Dmitry S. Kopitsyn, Maxim V. Gorbachevskii, Alexandra A. Kadykova, Alexander S. Shashkov, Marina V. Zhurina, Alexander I. Netrusov, Yuriy A. Knirel, Vladimir K. Plakunov, Marc G. J. Feuilloley

**Affiliations:** ^1^Winogradsky Institute of Microbiology, Federal Research Centre «Fundamentals of Biotechnology», Russian Academy of Sciences, Moscow, Russia; ^2^Zelinsky Institute of Organic Chemistry, Russian Academy of Sciences, Moscow, Russia; ^3^Department of Physical and Colloid Chemistry, Gubkin University, Moscow, Russia; ^4^Laboratory of Polymers, Biopolymers, Surfaces UMR 6270 PBS, Rouen University, Rouen, France; ^5^Higher Chemical College of the Russian Academy of Sciences, Mendeleyev University of Chemical Technology of Russia, Moscow, Russia; ^6^Department of Microbiology, Lomonosov Moscow State University, Moscow, Russia; ^7^EA4312 Laboratory of Microbiology Signals and Microenvironment, Rouen University, Evreux, France

**Keywords:** *Cutibacterium acnes*, biofilms, biofilm matrix, Orbitrap mass spectrometry, nuclear magnetic resonance, surface-enhanced Raman spectroscopy, polysaccharides, proteins

## Abstract

In skin, *Cutibacterium acnes* (former *Propionibacterium acnes*) can behave as an opportunistic pathogen, depending on the strain and environmental conditions. Acneic strains of *C. acnes* form biofilms inside skin–gland hollows, inducing inflammation and skin disorders. The essential exogenous products of *C. acnes* accumulate in the extracellular matrix of the biofilm, conferring essential bacterial functions to this structure. However, little is known about the actual composition of the biofilm matrix of *C. acnes*. Here, we developed a new technique for the extraction of the biofilm matrix of Gram-positive bacteria without the use of chemical or enzymatic digestion, known to be a source of artifacts. Our method is based on the physical separation of the cells and matrix of sonicated biofilms by ultracentrifugation through a CsCl gradient. Biofilms were grown on the surface of cellulose acetate filters, and the biomass was collected without contamination by the growth medium. The biofilm matrix of the acneic *C. acnes* RT5 strain appears to consist mainly of polysaccharides. The following is the ratio of the main matrix components: 62.6% polysaccharides, 9.6% proteins, 4.0% DNA, and 23.8% other compounds (porphyrins precursors and other). The chemical structure of the major polysaccharide was determined using a nuclear magnetic resonance technique, the formula being →6)-α-D-Gal*p*-(1→4)-β-D-Man*p*NAc3NAcA-(1→6)-α-D-Glc*p*-(1→4)-β-D-Man*p*NAc3NAcA-(1→3)-β-Gal*p*NAc-(1→. We detected 447 proteins in the matrix, of which the most abundant were the chaperonin GroL, the elongation factors EF-Tu and EF-G, several enzymes of glycolysis, and proteins of unknown function. The matrix also contained more than 20 hydrolases of various substrata, pathogenicity factors, and many intracellular proteins and enzymes. We also performed surface-enhanced Raman spectroscopy analysis of the *C. acnes* RT5 matrix for the first time, providing the surface-enhanced Raman scattering (SERS) profiles of the *C. acnes* RT5 biofilm matrix and biofilm biomass. The difference between the matrix and biofilm biomass spectra showed successful matrix extraction rather than simply the presence of cell debris after sonication. These data show the complexity of the biofilm matrix composition and should be essential for the development of new anti-*C. acnes* biofilms and potential antibiofilm drugs.

## Introduction

*Cutibacterium acnes*, a former member of the genus *Propionibacterium* (Scholz and Kilian, 2016), is one of the most abundant microorganisms found in the human skin. In some areas of the skin (pilosebaceous units), it is highly dominant and can represent up to 87% of all microorganisms (Fitz-Gibbon et al., 2013). The lifestyle of *C. acnes* is closely related to that of humans, and its abundant presence on the skin may be a consequence of the positive effect of such a coexistence (Christensen and Brüggemann, 2014). *C. acnes* are immobile, Gram-positive, facultative anaerobic, coryneomorphic, rod-shaped bacteria (Aubin et al., 2014), preferring sebaceous and moist skin areas (forehead, neck, and back) (Grice et al., 2009; Aubin et al., 2014; SanMiguel and Grice, 2015; Feuerstein et al., 2017). *C. acnes* can consume sebum due to the presence of lipases and is commonly found inside the hollows of the sebaceous glands and hair follicles (Lee et al., 1982). Some strains bearing a specific virulence plasmid are clearly associated with acne and considered to be acneic *C. acnes* (Fitz-Gibbon et al., 2013). However, the diseases associated with *C. acnes* are highly diverse. *C. acnes* is frequently found associated with a large variety of medical devices to which it can bind [shoulder implants (Patel et al., 2009), cardiac implants (Okuda et al., 2018), and contact lenses (Perry and Lambert, 2011)]. The frequency of shoulder implant infections due to *C. acnes* biofilms is particularly high (Zeller et al., 2007), presumably because *C. acnes* is common in these skin areas. The danger associated with the development of *C. acnes* on internal implants is due to the very long period between surgery and the appearance of clinical signs, up to several years (Uckay et al., 2010), because of the very slow growth of this microorganism in certain environments. *C. acnes* biofilms are also now considered to be one of the reasons for the stability of *C. acnes* during acne antibiotic treatment (Dessinioti and Katsambas, 2016).

The extracellular matrix is an integral feature of biofilms. It functions as a shield that protects bacteria from predators and participates in the formation of oxygen and signaling molecule gradients (McDougald et al., 2012). In addition, the matrix protects the bacteria against antibiotics and host immune defense factors (Huang et al., 2011). It is generally considered that the bacterial biofilm matrix consists of polysaccharides, proteins, and extracellular nucleic acids (Dogsa et al., 2013). The functions attributed to the biofilm extracellular matrix to date include structure, sorption, surface activity, catalytic activity, information, redox activity, and nutrition (Marvasi et al., 2010). The biofilm matrix acts as a strong barrier against various active compounds (Zhurina et al., 2014), and it is well demonstrated that bacteria in biofilms are particularly resistant to antibiotics and biocides. This is a major problem for the eradication of pathogens, and in this light, determining the matrix composition is a key element for the development of compounds with antibiofilm activity. However, although the target is simple to define, the goal is technically difficult to achieve. Indeed, bacteria and matrix molecules are intimately associated with the growth medium, and it is particularly difficult to extract matrix compounds without inducing massive bacterial lysis or without artifacts due to contamination by the growth medium.

*Cutibacterium acnes* biofilms are, thus, of major interest, but our knowledge of their structure and matrix composition is still marginal. Most previous research of extracellular polymers of *C. acnes* focused on suspensions or planktonic cells and their properties, not biofilms. Differences in the ability of *Cutibacterium* strains to form extracellular polymers were shown. Mak et al. (2013) showed varying ability of former cutaneous *Propionibacterium* species (*Propionibacterium avidum*, *Propionibacterium granulosum*, and *Propionibacterium acnes*) to form extracellular polysaccharides or pili-like structures that was likely due to differences in the genomes of the bacteria. Cells of *P. acnes* strains 266 and KPA171202 grown in liquid medium did not have polysaccharide-based extracellular structures, and cells of *P. avidum* strains had rich polysaccharide envelopes surrounding the cells (Mak et al., 2013). Recently, a new extracellular antioxidant protein, RoxP, of *C. acnes* was described (Allhorn et al., 2016), and its expression appears to be strain specific (Andersson et al., 2019). RoxP is abundantly secreted into liquid culture medium and can have a beneficial role for host cells and *C. acnes* in skin (Allhorn et al., 2016; Andersson et al., 2019). None of these studies focused on the biofilm properties of *C. acnes*. This is critical for two reasons. The first is that biofilms of *C. acnes* are usually formed in various niches: on prosthetic joint implants (Holmberg et al., 2009) or in skin glands and hair follicles (Jahns and Alexeyev, 2014). The second is that the biofilm phenotype results from changes in the expression of a multitude of genes (Nikolaev and Plakunov, 2007) and starts when microbial cells settle on the surface (Belas, 2014). It is, thus, crucial to study true biofilms to understand their extracellular matrix. Indeed, the extrapolation of the properties of planktonic cells to biofilms is debatable. There are a few studies dedicated to the matrix of *C. acnes*. The first showed that the matrix of *C. acnes* biofilms contains DNA, proteins, and polysaccharides (Jahns et al., 2016). This already complements the work of Mak et al. (2013), in which no extracellular polysaccharides of *C. acnes* cells were found. The second study focused on seven strains of *C. acnes* isolated from the biofilms on cardiac pacemakers and showed poly-*N*-acetylglucosamine (PNAG) to be a polysaccharide component of the matrix (Okuda et al., 2018). However, these studies raised a number of methodological concerns because, as previously mentioned, obtaining a clean biofilm matrix is difficult. There is also probably certain strain-related variability and the appearance of ribotyped *C. acnes* strains as references made it necessary to study this problem.

## Materials and Methods

### Growth of the *C. acnes* RT5 Biofilm and Preparation of the Biofilm Biomass for Matrix Extraction

Three-day cultures of *C. acnes* RT5 in reinforced clostridial medium (RCM; pH 7.0) were washed twice with sterile physiological saline (pH 7.0), and the OD_580_ was adjusted to 0.5. RCM agar (25 mL) was distributed into 90-mm petri dishes (PDs). The composition of RCM in distilled water was (g/L) yeast extract, 13.0; peptone, 10.0; NaCl, 5.0; glucose, 5.0; sodium acetate, 3.0; soluble starch, 1.0; and L-cysteine hydrochloride, 0.5. Solid RCM agar contained 1.5% agar. Two sterile hydrophilic 25-mm cellulose acetate filters (Sartorius) were placed in each PD. These filters were used as carriers to allow biofilm formation and biomass production in the absence of contamination by the medium. Then, 200 μL of a washed cell suspension was placed on each filter and distributed over the entire filter surface with a pipette tip. A total of 14 PDs with filters were incubated together in a GasPack^®^ anaerobic chamber at 37°C for 7 days. After incubation, 1.5 mL wet biomass was harvested into a 50-mL Falcon conical centrifuge tube (Corning), mixed with 1 mL of sterile milliQ (MQ) water, and homogenized by vortexing. After a first round of vortexing, the biomass was centrifuged for 5 min at 2,500 × *g* at room temperature (RT). *C. acnes* is considered to be very resistant to sonication because of its cell wall structure and according to data obtained for certain Gram-positive and Gram-negative bacteria (Monsen et al., 2009). The matrix was separated from the bacterial cells by sonicating the biomass either in a Branson Digital Sonifier 250 (United States), with 3/16″ exponential peaks for 15 min with an amplitude of 25% (120 μm) and a frequency of 20 kHz, or a Sonyprep 150 Plus (Great Britain), with 3-min exponential peaks for 10 min at a frequency of 23 kHz and an amplitude of 150 μm.

### *C. acnes* RT5 Matrix Extraction

The matrix was then extracted from the biomass by ultracentrifugation on a cesium chloride gradient. The gradient was prepared in a 26.3-mL Beckmann Coulter 355618 PC ultracentrifuge tube by the addition of 3 mL of ω 46%, 28%, 14%, and 7% CsCl solutions (Table 1). Then, the suspension of the sonicated biomass was layered onto the top of the CsCl gradient and centrifuged for 3 h at 170,000 × *g* using a Beckmann Coulter Optima X-100 ultracentrifuge and an 70Ti rotor. After centrifugation, the supernatant containing the matrix was collected and distributed into Corning centrifuge tubes and centrifuged for 20 min at 15,000 × *g* to remove any cell residues from the liquid after supernatant sampling from the tube. The OD_540_ of the matrix samples was measured, and the samples were stored at −20°C.

**Table 1:** CsCl solutions used in the gradient column (in order of layering).

No. of CsCl solution	ω (CsCl), %	$d_{20}^{20}$, g/mL
1	46	1.5158
2	28	1.2644
3	14	1.1163
4	7	1.054

### Evaluation of Bacterial Lysis by Measuring Lactate Dehydrogenase (LDH) Activity

Lactate dehydrogenase (LDH) is an intracellular enzyme (Chao et al., 1988; Lin et al., 2018) and can, thus, be employed as an indicator of cell lysis during biofilm matrix extraction, as *C. acnes* constitutively expresses LDH (Counotte et al., 1980; Koniarova, 1993; Brzuszkiewicz et al., 2011). LDH activity was measured using a commercial kit (CytoTox 96^®^ Non-Radioactive Cytotoxicity Assay, Promega, United States). Briefly, the method is based on the colorimetric measurement of formazan (OD_490_) formed during the conversion of lactate into pyruvate by LDH. In this reaction, NADH is the source of electrons used for the transformation of iodide–tetrazolium violet into formazan. *C. acnes* RT5 from centrifugation pellets was used as positive control. The cell suspension was adjusted to the level of the matrix suspension optical density (OD) and the LDH activity measured in both compartments.

### Complementary Biofilm Extraction Tests

The stability of *C. acnes* RT5 during biofilm matrix extraction was evaluated by subjecting the biomass, containing cells and matrix, to more harsh conditions, i.e., complementary treatment with lysis buffer from the LDH assay kit (Promega, United States), lysozyme (Sigma), according to Nagaoka et al. (1985), and even association with sonication (Table 2). After centrifugation, as described elsewhere, the LDH activity was measured in the supernatant (extracellular compartment). In parallel, an aliquot of each sample was layered onto a microscopic slide, fixed with 96% ethanol, and stained with crystal violet (CV; 0.1%). The presence of bacterial cell bodies or fragments was examined by microscopy using a Zeiss Axio Observer A1 microscope.

**Table 2:** Complementary biofilm extraction conditions tested (by order of harshness increment).

Condition no.	Description
1	Intact biomass without treatment (control)
2	Biomass was treated with 1X lysis buffer (Promega) for 40 min at RT
3	Biomass was treated with 0.01 mg/mL lysozyme in 1X lysis buffer (Promega) for 30 min at 37°C
4	Cells were treated with 1 mg/mL lysozyme in buffer (0.1 M tris-HCl + 0.05 M ethylenediaminetetraacetic acid (EDTA), pH 8.0) for 30 min at 37°C. Then, the biomass was sonicated in a Branson Digital Sonifier 250 (United States) with 3/16″ exponential horn for 15 min at 50% amplitude (229 μm) and a frequency of 20 kHz
5	Cells were treated with 2 mg/mL lysozyme in buffer (0.1 M tris-HCl + 0.05 M EDTA, pH 8.0) for 30 min at 37°C. Then, the biomass was precipitated and the pellet resuspended in 5X lysis buffer (Promega) and incubated for 40 min at RT. Then, the biomass was sonicated in a Branson Digital Sonifier 250 (United States) with 3/16″ exponential horn for 15 min at 50% amplitude (229 μm) and a frequency of 20 kHz in the presence of glass beads

### Quantification of Total Organic Carbon in the *C. acnes* RT5 Biofilm Matrix

The total organic carbon content of the matrix was measured using a wet mineralization method. Organic matter was mineralized by an adapted method (Walkley, 1947) using a mix of concentrated sulfuric acid (SA; Sigma) and potassium dichromate (Sigma). Briefly, 14.71 g of dichromate was dissolved in 150 mL of MQ water and 100 mL of 96% SA was added. Matrix samples were diluted 10 times, and 1 mL of each diluted sample was mixed with 3 mL of reagent mix and incubated in a boiling water bath (100°C). In parallel, a series of glucose solutions of various concentrations (10–2,000 μg/mL in MQ water) were prepared to make calibration curves. After boiling for 90 min, the samples were cooled and the OD_590_ was measured. The total organic carbon content was quantified on the basis of a carbon mass proportion of 40% in glucose (the molecular mass of glucose is 180 g/mol, of which carbon is 72 g/mol).

### Quantification of Reducing Sugars and Proteins in the Matrix

The concentration of reducing sugars, as a marker of total carbohydrates, was measured using the anthrone method (Dreywood, 1946). SA reacts with carbohydrates, and the resulting furfurols condensate with anthrone, with the production of colored compounds. Briefly, 0.2 g of anthrone (Sigma) was dissolved in 100 mL of 96% SA (Sigma) to make the anthrone reagent. Standard glucose solutions were prepared for calibration curves. One gram of glucose (Sigma) was dissolved in 100 mL of a benzoic acid solution made by dissolving 2.5 g of benzoic acid in 1 L of MQ water. Standard glucose solutions were stored at 4°C in the dark. Before analysis, partial hydrolysis of the polysaccharides was performed to increase the concentration of reducing sugar groups. Matrix samples were diluted 50- to 80-fold in MQ water. Two milliliters of each diluted sample were mixed with 200 μL of 96% SA in glass tubes and then heated for 4 h in a boiling water bath. After hydrolysis was complete, the samples were cooled to RT and a double volume of anthrone reagent was added to each sample. Samples were mixed and heated again at 100°C for 16 min. In parallel, the same reaction was performed with a series of glucose solutions (made from a standard solution), with the concentration ranging from 5 to 50 μg/mL. MQ water was used as a negative control. After incubation, the tubes were cooled to RT and the OD_625_ was estimated. Finally, the concentration of the reducing sugars was calculated using the calibration curve.

Proteins were quantified using the Bradford method as described in Bradford (1976). Standard Bradford reagent (Sigma) and bovine serum albumin (BSA, Sigma) were used.

### Detection of DNA Concentration in the *C. acnes* RT5 Matrix

The DNA concentration in the matrix was estimated using the Dische method (Dische, 1955). This method is based on the reaction of desoxyribose with diphenylamine in acetic acid. When heated, levulinic aldehyde is produced and condenses with diphenylamine to produce a colored compound. Briefly, the diphenylamine solution was prepared by dissolving 1 g of diphenylamine (Sigma) in 100 mL of pure acetic acid (Emsure, Germany). Then, 2.75 mL of 96% SA was added. A calibration series of standard DNA solutions (50–500 μg/mL) was then prepared using herring sperm DNA (Sigma), and the matrix samples were prepared for analysis. All samples (2 mL) were distributed into glass tubes. Then, a double amount of Dische reagent (4 mL) was added, and the samples were heated at 100°C in a water bath. The OD_595_ was measured after cooling. MQ water was used as a control.

### Orbitrap Mass Spectrometry (MS) Investigation of the *C. acnes* RT5 Matrix Proteome

Protein samples from the matrix were first prepared for Orbitrap MS. First, matrix samples were dialyzed in 0.1- to 0.5-kDa pore size bags (Spectrum Repligen, United States) to remove the CsCl. Then, the samples were mixed with a 4× volume of acetone and incubated for 10 min at RT to precipitate the proteins. After precipitation, the samples were centrifuged at RT at 15,000 × *g* for 30 min to collect the proteins. A Bradford assay was performed to follow the protein content and determine potential loss at all steps (before dialysis, after dialysis, liquid supernatants after protein removal). The remaining liquid supernatants were transferred into 140-mm PD, and the acetone was evaporated at RT for 30 min. This remaining liquid without acetone was also tested for protein content by Bradford assay.

Direct preparation of the samples for analysis consisted of the following steps. Proteins were resuspended in 1 mL of acetone, transferred to Eppendorf tubes, and precipitated by centrifugation at 13,000 × *g* for 10 min at RT. The supernatant was then removed, and the samples were dried in a SpeedVac evaporator for 15 min. If necessary, samples were stored at −20°C. Before analysis, the proteins were dissolved in 25 μl of R2D2 buffer consisting of 7 M urea, 2 M thiourea, 5 mM tri-*n*-butylphosphine, 20 mM dithiothreitol, 0.5% 3-(4-hepty)phenyl-3-hydroxypropyl dimethylammonium propyl sulfonate (C_7_BzO), and 2% 3-[(3-cholamidopropyl) dimethylammonium]-1-propylsulfate hydrate (CHAPS). The proteins were then separated, digested with trypsin, and subjected to MS.

Protein samples were mixed with sodium dodecyl sulfate (SDS) loading buffer [63 mM tris-HCl, pH 6.8, 10 mM dithiothreitol (DTT), 2% SDS, 0.02% bromophenol blue, 10% glycerol] and loaded onto an SDS-polyacrylamide gel electrophoresis (PAGE) stacking gel (7%). A short electrophoresis was then performed (10 mA, 15 min). After migration, the gels were stained with Coomassie blue and destained with a solution containing 50% ethanol, 10% acetic acid, and 40% deionized water. The revealed protein bands were excised, washed with water, and digested with trypsin (2 μg per band) overnight at 37°C. Several steps of peptide extraction were performed in H_2_O/acetonitrile/trifluoroacetic acid (49.5/49.5/1). The peptides were then dried and stored at −20°C. For tandem MS, all experiments were performed on an LTQ Orbitrap Elite (Thermo Scientific) coupled to an Easy nLC II system (Thermo Scientific). One microliter of sample was injected into an enrichment column (C18 PepMap100, Thermo Scientific). The separation was performed with an analytical column needle (NTCC-360/100-5-153, Nikkyo Technos, Japan). The mobile phase consisted of H_2_O/0.1% formic acid (FA) (buffer A) and CH_3_CN/FA 0.1% (buffer B). Tryptic peptides were eluted at a flow rate of 300 nL/min using a three-step linear gradient: from 2 to 40% B over 75 min, from 40 to 80% B over 4 min, and 11 min at 80% B. The mass spectrometer was operated in positive ionization mode with the capillary voltage and source temperature set at 1.5 kV and 275°C, respectively. The samples were analyzed using the CID (collision-induced dissociation) method. The first scan (MS spectra) was recorded in the Orbitrap analyzer (*R* = 60,000) with a mass range of *m*/*z* 400–1,800. Then, the 20 most intense ions were selected for MS^2^ experiments, from which the singly charged species were excluded. Dynamic exclusion of already fragmented precursor ions was applied for 30 s, with a repeat count of 1, a repeat duration of 30 s, and an exclusion mass width of ±10 ppm. Fragmentation occurred in the linear ion trap analyzer with a collision energy of 35 eV. All measurements in the Orbitrap analyzer were performed with on-the-fly internal recalibration (lock mass) at *m*/*z* 445.12002 (polydimethylcyclosiloxane). Raw data were then imported to Progenesis LC-MS software (Nonlinear Dynamics). For comparison, one sample was set as a reference, and the retention times of all other samples within the experiment were aligned. After alignment and normalization, statistical analysis was performed for one-way analysis of variance (ANOVA) calculations. Only peptide features with a *p*-value < 0.05, *q*-value < 0.05, and power > 0.8 were retained for quantification. MS/MS spectra from selected peptides were exported for peptide identification with Mascot (Matrix Science) used against the database restricted to *C. acnes*. Database searches were performed with the following parameters: one missed trypsin cleavage site allowed; variable modifications: carbamidomethylation of cysteine and oxidation of methionine. For each growth condition, the total cumulative abundance of the protein was calculated by summing the abundances of the peptides. Proteins with a *p*-value < 0.05, *q*-value < 0.05, and power > 0.8 were retained. Moreover, only proteins for which the average normalized abundances between growth conditions differed by more than twofold were retained.

### Surface-Enhanced Raman Spectroscopy of the *C. acnes* RT5 Matrix

Surface-enhanced Raman spectroscopy is a powerful method for the non-destructive study of organic samples, particularly bacterial biofilms (Chao and Zhang, 2012). With suitable active substrates and near-infrared excitation, it is possible to register Raman signals of characteristic bacterial substances [nicotinamide adenine dinucleotide (NAD)-like redox complexes, proteins, etc.] (Efrima and Zeiri, 2009) and discriminate between bacteria, at least at the genus level (Kopitsyn et al., 2019). Surface-enhanced Raman scattering (SERS) substrates were prepared as described elsewhere (Gorbachevskii et al., 2018; Kopitsyn et al., 2019).

Surface-enhanced Raman scattering was used in this study to obtain the spectra of *C. acnes* biofilms and verify successful matrix extraction. The intact biomass of biofilms, cell biomass after matrix extraction, and isolated biofilm matrix of *C. acnes* RT5 were used for SERS analysis. The biomass (intact and after matrix extraction) of *C. acnes* RT5 was washed three times with acetone and dried at RT. Before SERS analysis, dry samples were washed twice with a 0.9% NaCl solution. The pellets were resuspended, centrifuged at 3,000 × *g*, and kept at RT for 10 min before analysis.

Nanoparticles were characterized by transmission electron microscopy (TEM) and scanning electron microscopy (SEM). For TEM, 5 μL of a nanoparticle suspension was placed on a TEM grid (Ted Pella, Redding, CA, United States) and dried at RT. TEM images were obtained using a JEM-2100 microscope (Jeol) with an acceleration voltage of 200 kV. SEM images were obtained using a JIB-4501 microscope (Jeol).

Surface-enhanced Raman scattering spectra were registered using a BWS415 spectrophotometer (BWTEK). Samples were placed on an XYZ stage, and the laser position was controlled *via* a USB-connected microscope Mikmed-2000R (Micromed). Various substrates based on gold nanomaterials were tested to register the SERS signals.

### Isolation of Cell Wall Polysaccharides

A cell wall preparation was obtained by disintegrating the bacterial cells using a UZDN_1 ultrasonic disintegrator as described previously (Streshinskaya et al., 1979). Polysaccharides were extracted from the cell walls with 10% trichloroacetic acid (TCA) (1:10 w/v) at 4°C for 48 h. The mixture was centrifuged and the supernatant was dialyzed against distilled water in SERVAPOR bags with a pore size of 12 kDa and lyophilized to generate the cold extract. The centrifugation pellet was extracted with 10% TCA at 100°C for 5 min, and the supernatant was dialyzed against distilled water and lyophilized to generate the hot extract. Both preparations were dissolved in NaOAc buffer and applied to a column (80 × 1.6 cm) of TSK HW-40 (S) using 1% AcOH as eluent and monitored with a Knauer differential refractometer.

### Isolation of Matrix Polysaccharides

The matrix of *C. acnes* RT5 was dialyzed against distilled water in small-pore bags. After dialysis, an aliquot of trichloroacetic acid was added to the matrix sample to a final pH of 2.0 for nucleic acid and protein sedimentation. The extraction and other procedures were the same as those already described for that of the cell wall polysaccharides.

### Sugar Analysis

Hydrolysis was performed with 2 M CF_3_CO_2_H (120°C, 2 h). Monosaccharides were identified as the alditol acetates (Sawardeker et al., 1965) by gas-liquid chromatography (GLC) on an HP-5 capillary column using a Maestro (Agilent 7820) chromatograph (Interlab, Russia) and a temperature gradient of 160°C (1 min) to 290°C at 7°C min^−1^.

### Nuclear Magnetic Resonance (NMR) Spectroscopy of Sugars

Samples were deuterium exchanged by freeze drying twice from 99.9% D_2_O and then examined as solutions in 99.95% D_2_O. ^1^H and ^13^C NMR spectra were recorded on a Bruker Avance II 600 MHz spectrometer (Germany) at 45°C using internal sodium 3-trimethylsilylpropanoate-2,2,3,3-d4 (δ_H_ 0.0, δ_C_ −1.6) as a reference. Bruker TopSpin 2.1 software was used to acquire and process the NMR data. A spin lock time of 60 ms and a mixing time of 200 ms were used for the total correlation spectroscopy (TOCSY) and rotating-frame Overhauser spectroscoPY (ROESY) experiments, respectively. A ^1^H,^13^C heteronuclear multiple bond correlation (HMBC) experiment was performed with a 60-ms delay to study the evolution of long-range couplings.

The ^13^C NMR chemical shifts were analyzed and compared to published values by GODDESS (Kapaev and Toukach, 2016) and GRASS NMR (Kapaev and Toukach, 2018) simulation, and structure elucidation web services were implemented at the platform of the Carbohydrate Structure Database (Toukach and Egorova, 2016).

### Statistics

Experiments were independently repeated at least five times. Data were processed using Microsoft Excel software. All numeric data were analyzed using the Mann–Whitney non-parametric test. Numeric results are presented as average means with the standard error of the mean as error bars.

## Results

### Matrix Extraction of *C. acnes* RT5

**FIGURE 1:**
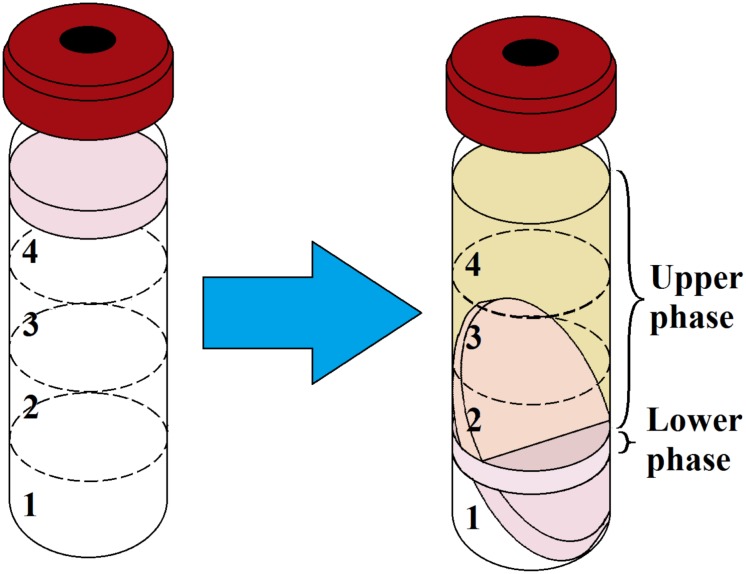
Ultracentrifugation of prepared *C. acnes* RT5 biofilm biomass and matrix extraction. **(Left)** Gradient formation and inoculation of the sonicated biomass. **(Right)** Typical aspect of the two phases of the matrix after ultracentrifugation. Nos. 1, 2, 3, and 4 refer to the CsCl solutions as indicated in Table 1.

The average biomass of the biofilm was 1.5 cm^3^ or 0.46 g after a week of incubation. After matrix extraction and ultracentrifugation, the supernatant containing the matrix was split into two phases (Figure 1): a brownish (because of porphyrins), transparent supernatant upper phase (SUP) and a turbid supernatant lower phase (SLP). The division of the SUP and SLP was solely a result of the matrix extraction protocol and was not associated with the spatial structure of the biofilm matrix *in vivo*. The average OD_540_ of the upper and lower phases was 0.1 and 1.2, respectively. The average volume was 12 ± 0.5 mL for the SUP and 3 ± 0.5 mL for the SLP. We carried out the quantitative biochemical analysis, proteomic analysis, and SERS for the SUP and SLP separately because of differences in their composition. For quantitative biochemical analysis, we also analyzed the total matrix supernatant (TMS). TMS calculations were based on the volumes and concentrations of the compounds in each phase.

Measurement of the LDH activity showed the OD_490_ of formazan to be approximately 0.05 for both the SUP and SLP. The OD_490_ for samples of control intact bacteria was 0, whereas it reached 0.1 in bacterial suspensions treated with lysis buffer. The highest measured LDH activity was obtained in biomass samples after ultracentrifugation without additional treatment (intact), whereas the OD_540_ of the suspensions was 1.0. The OD_490_ of formazan was approximately 1.5 for the same samples. This shows that the basal LDH activity of *C. acnes* RT5 is low. During the growth of the biofilm, a small portion of the cell population continually dies, and their contents diffuse into the matrix, explaining the small amount of LDH detected in the matrix samples.

**FIGURE 2:**
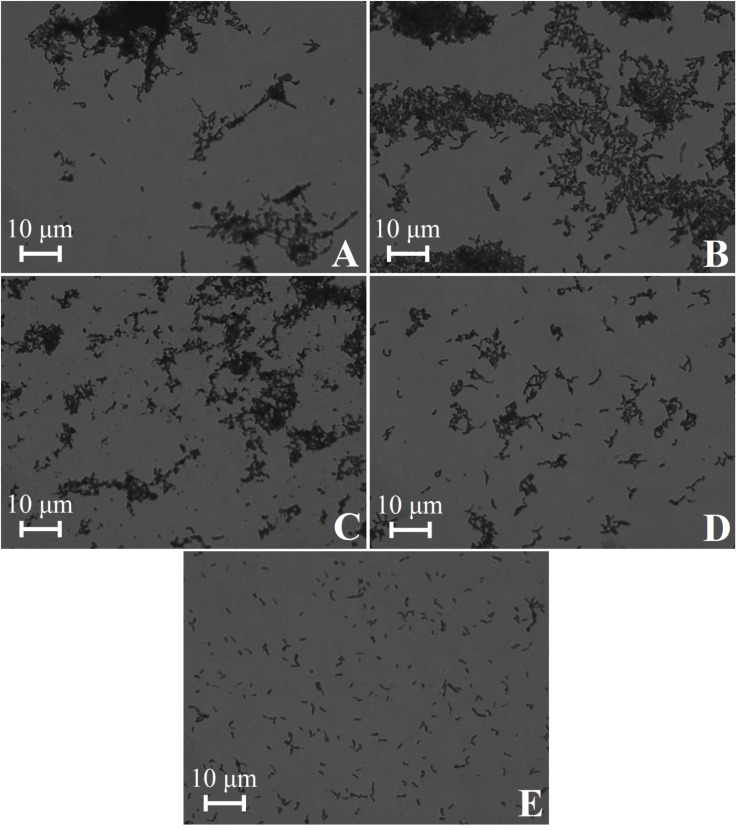
Light microscopy of *C. acnes* RT5 cell suspensions treated according to various lytic protocols. **(A)** Intact cells (control). **(B)** Lysis buffer at room temperature (RT). **(C)** Lysozyme (0.01 mg/mL) + lysis buffer at 37°C. **(D)** Lysozyme (1 mg/mL) + lysis buffer + ultrasound. **(E)** Lysozyme (2 mg/mL) + lysis buffer + ultrasound in the presence of glass beads. See Table 2 for further details.

The *C. acnes* RT5 cells were treated with various lytic agents (Table 2) to verify that the bacteria were not critically damaged during the matrix preparation. In all the cases, the OD_490_ measured in the LDH assay was approximately 0.1, indicating that the membranes of *C. acnes* were not damaged even after exposure to lysozyme and ultrasound. Moreover, cell aggregates were not dispersed, as observed by light microscopy (Figure 2A–C), except after exposure to the harshest conditions (lysozyme + ultrasound + glass beads) (Figure 2E). Cell aggregates were also partially dispersed if the bacteria were exposed to a combination of 1 mg/mL lysozyme and ultrasound, but without glass beads (Figure 2D).

### Quantitative Biochemical Analysis of the Basic Matrix Components of *C. acnes* RT5 Biofilms

We quantified the proportion of the basic *C. acnes* RT5 matrix components extracted by our method using biochemical methods. We did not calculate the dry mass of the matrix for several reasons. The presence of CsCl in the solutions made it impossible to determine the dry weight of the matrix, and the use of dialysis bags, even with the smallest pore size, probably led to the loss of organic matter [especially low-molecular-weight (LMW) compounds]. Variations of the phase volumes were small. Thus, we first calculated the amount of matrix components as mg/mL of liquid volume and then μg/mg of biofilm mass.

The calculations were based on the total organic carbon content, and the results are summarized in Table 3. The total mass of organic carbon in the extracted biofilm matrix was approximately 31.5 mg or 68.47 μg/mg of dry biofilm biomass. The concentration of organic carbon was more than twofold higher in the SUP than in the SLP, probably because of the presence of high amounts of LMW organic compounds (pigments and metabolites). The SLP consisted mainly of high-molecular-weight (HMW) molecules (proteins and DNA) and did not contain as much pigment as the SUP.

**Table 3:** Composition of the organic material of the extracted *C. acnes* RT5 biofilm matrix.

	Reducing sugars		Proteins		DNA		Other organics	Total organic carbon
	μg/mL	%	μg/mL	%	μg/mL	%	%	μg/mL
SUP (12 mL volume)	3,717.1 ± 755.9	66.7 ± 13.6	307.6 ± 61.2	6.9 ± 1.4	139.8 ± 21.4	3.1 ± 0.5	23.3 ± 15.5	2,229.1 ± 426.7
SLP (3 mL volume)	1,444.3 ± 462.3	38.5 ± 12.3	531.8 ± 248.7	25.9 ± 12.1	274.9 ± 76.0	9.1 ± 2.5	26.5 ± 26.9	1,501.8 ± 364.3
TMS (15 mL volume)	3,262.5 ± 687.5	62.6 ± 13.2	401.4 ± 96.5	9.6 ± 2.3	166.8 ± 28.3	4.0 ± 0.7	23.8 ± 16.6	2,083.6 ± 406.4

We calculated the average proportion of carbon in the molecular mass for each matrix component. For carbohydrates, the proportion of carbon in glucose corresponds to 40% of the molecular mass, and for proteins, it is 45% based on the proportion of carbon in BSA. For DNA, we used an average proportion of carbon in 100 bp of *C. acnes* DNA, considering a G–C pair ratio of approximately 60% (Brzuszkiewicz et al., 2011). This led to a value of 32%. Carbohydrates were the most abundant component in the matrix for both phases, especially the SUP. The concentration of reducing sugars was significantly lower in the SLP, probably due to their molecular weight and distribution in the CsCl gradient. Polysaccharides generally represented more than 60% of the organic material in the TMS.

The second most abundant component of the *C. acnes* RT5 matrix was protein. Here, the situation was opposite to that of the polysaccharides. The protein concentration was approximately twofold lower in the SUP than in the SLP. Although the HMW proteins were probably more abundant in the SLP than in the SUP, their total amount was less than that of the LMW proteins because of the differences in volume (an average of 3 mL SLP *versus* 12 mL SUP). The calculated total mass of protein of the extracted matrix was approximately 26.67 μg/mg of the dry biofilm biomass.

DNA was the polymer with the lowest concentration in the extracted matrix. Its concentration in the SUP was twofold less than that in the SLP. We calculated the ratio of the LMW to HMW DNA molecules in the matrix to be 2:1, based on its spatial distribution in the CsCl gradient and concentrations in the SUP and SLP. The total mass of DNA was approximately 2.5 mg per 1.5 cm^3^ of wet biomass or 5.43 μg/mg of dry biofilm biomass. The low concentration of DNA in the matrix shows that our matrix extraction method did not damage or destroy the cells. Indeed, control experiments carried out using the same technique on a biofilm of 0.1 cm^3^ of a transformed strain of *Escherichia coli* ET12567 showed that the only DNA obtained was that of the plasmid pTetONCFPOpt (Sastalla et al., 2009) at a concentration of 100 μg/mL of the extract (*data not shown*).

We performed wet combustion and calculated the proportion of all major polymers in the *C. acnes* RT5 matrix. Approximately 23.8% of the organic carbon found in the TSM was not from peptides, sugars, or DNA, accounting for 23.3% of the total organic carbon in the SUP and 26.5% in the SLP (Table 3). Given the metabolism of *C. acnes*, it is likely that these compounds represent various metabolites, including essentially porphyrins and their precursors. Indeed, a qualitative reaction showed a high amount of α-aminolevulinic acid (*data not shown*).

### Proteomics Analysis of *C. acnes* RT5 Matrix

We performed Bradford tests to control for protein loss at each step of the preparation for Orbitrap analysis. Before dialysis, we detected 19.5 μg/mL protein in the SUP and 116.45 μg/mL in the SLP. After dialysis, the protein content of the SUP was 14.3 and that of the SLP was 61.0 μg/mL, corresponding to a protein mass of 28.57 and 305.19 μg, respectively. Thus, there was high protein loss (−47.6%) from the lower phase during dialysis. Given the pore diameter of the dialysis membrane, such loss was probably due to that of very short-chain peptides.

Orbitrap proteomics analysis of the *C. acnes* RT5 biofilm matrix showed a total of more than 400 different proteins (see Supplementary Table S1). Many were catalytic enzymes involved in the metabolism of sugars, proteins, and nucleotides. Many of the proteins found in the matrix extract were components (domains or monomers) of more complex molecular structures, such as ribosomes and cytochromes. The presence of many intracellular enzymes can be explained by normal cell autolysis that occurs during the formation and maturation of the biofilm. The same was true for non-enzymatic proteins. Overall, we found proteases, nucleases, and enzymes involved in carbohydrate processing, and 48 hypothetical proteins with unknown function. We also found 43 ribosomal proteins in the biofilm matrix (one is putative).

There were slight differences in the 30 most abundant proteins found in the SUP and SLP, but 22 were the same (Supplementary Table S2). The dominant protein of the *C. acnes* RT5 matrix was chaperonin GroL; it was the most abundant protein in both the SUP and SLP. Chaperonin GroS was also among the most abundant proteins, along with the chaperone DnaK. Chaperonin GroL is a homolog of the mammalian Hspd1 protein, which has been annotated to bind DNA^1^. In *E. coli*, this protein is normally found in the cytoplasm, but it can also be found in the membranes (Li and Young, 2012). The protein HMPREF9571_00996, of unknown function, was the second most abundant protein in the SUP and the fifth in the SLP. Five of the 30 the most abundant proteins of the matrix have no known function. They may be a part of the structural component of the biofilm matrix or perform other functions. A DoxX family protein of 210 amino acids was present in the SUP. The DoxX family proteins include transmembrane proteins of unknown function, probably involved in the regulation of the sodium balance in axonal membranes in eukaryotes (Tran et al., 2010). The functions of DoxX proteins in *C. acnes* RT5 biofilms are not clear. The elongation factors EF-Tu and EF-G were also abundant in both the SUP and SLP. The presence of EF-Tu, a normally highly abundant protein in cells (Weijland et al., 1992) may be the result of normal cell autolysis during biofilm formation. However, EF-Tu has recently been reported to be a moonlighting protein, which may act as a sensor of substance P in Gram-positive bacteria (Mijouin et al., 2013). Thus, these factors may also play another role in biofilms. The same may be true for enzymes of the glycolysis and propionic acid fermentation pathways, ribosomal proteins, domains of RNA polymerase domains, ATP synthases, and other enzymes (Table 4) found to be among the 30 the most abundant proteins of the matrix. Enolase was also found to be an abundant protein in a previous study (Okuda et al., 2018), and its function in the matrix requires further study.

**Table 4:** ^1^H and ^13^C nuclear magnetic resonance (NMR) chemical shifts (δ, ppm).

Sugar residue	H-1	H-2	H-3	H-4	H-5	H-6
	C-1	C-2	C-3	C-4	C-5	C-6
→6)-duD-Gal*p*-(1→ (**A**)	5.14	3.69	3.74	3.91	3.95	3.73/3.89
	99.8	69.5	70.4	70.0	71.5	68.1
→6)-/3D-Glc*p-*(1→ (**B**)	5.08	3.35	3.59	3.41	3.73	3.88/3.98
	99.8	72.7	74.1	70.4	72.3	69.3
→4)-β-D-Man*p*NAc3NAcA-(1→(**C**)	4.99	4.30	4.29	3.94	3.88	
	101.3	52.9	54.5	79.7	72.9	175.9
→4)-β-D-Man*p*NAc3NAcA-(1→ (**D**)	4.89	4.41	4.32	3.96	3.90	
	101.1	52.6	54.3	79.6	73.0	176.0
→4)-β-D-Gal*p*NAc-(1→ (**E**)	4.64	3.97	3.85	4.14	3.73	3.76
	102.7	52.0	82.3	68.2	76.2	62.5

Chemical shifts for NAc are at δ_H_ 1.97 and 2.01, δ_C_ 23.4 and 175.4 and 175.8 (for residues C and D, respectively); δ_H_ 2.07, δ_C_ 23.4 and 176.4 (for residue E). In addition, we found three proteins of the DoxX family (270, 210, and 133 amino acids in length) in the *C. acnes* matrix. We also found several established and putative hydrolases for various substrates, in particular two cellulases, another glycosyl hydrolase, nucleotide hydrolases, and an amidohydrolase, which could all play a role in biofilm formation and substrate consumption during biofilm formation in the skin. In particular, glycerophosphodiester phosphodiesterase, an enzyme involved in lipid metabolism, may be an important factor in the establishment of *C. acnes* RT5 in the skin, as well as its pathogenicity. Putative HAD hydrolase, which was found in the *C. acnes* RT5 matrix, may also be involved in lipid cleavage (Caparrós-Martín et al., 2013), as well as another putative hydrolase. Other possible hydrolases found in the matrix may also be involved in lipid metabolism (see Supplementary Table S1). In addition to hydrolases, we found a number of enzymes involved in various intracellular processes in the matrix of *C. acnes* RT5: sugar catabolism, transcription and translation, cell cycling, and amino acid synthesis. We also identified initiation, elongation, and termination transcription factors (see Supplementary Table S1). Finally, catalase, peroxiredoxin, and superoxide dismutase (SOD) were also present in the matrix and may participate in protection against oxidative stress.

We also found the putative streptolysin-associated protein SagB in the matrix of *C. acnes* RT5. SagB is a 343-amino-acid protein. For comparison, SagB of *Streptococcus pyogenes* (AF067649) contains 316 amino acids^1^. In the *Streptococcus* genus, SagB is a cytoplasmic cyclodehydrogenase involved in streptolysin SagA processing using flavin mononucleotide as an electron acceptor (Molloy et al., 2011). In *C. acnes* RT5, this putative protein is likely involved in similar processes of hemolysin metabolism that are important for pathogenicity. We also found the putative chaperone FliS in the lower phase. This protein is involved in flagella synthesis in various motile bacteria (Altegoer et al., 2018). The role of a putative FliS in a non-motile bacterium, such as *C. acnes*, is not clear, but it could have other functions possibly directly connected with the composition of the protein portion of the biofilm matrix, because it requires extracellular folding and post-translation modification. *C. acnes* normally contains five Christie, Atkins, Munch-Peterson (CAMP) factors (Valanne et al., 2005). We did not find any CAMP factors in the matrix, but rather proteins that are very similar to them. For example, hypothetical protein HMPREF9571_02536 shares more than 99% similarity with the CAMP-1 factor of other strains of *C. acnes* (according to the Uniprot^2^ and NCBI^3^ databases). It consists of a 285-amino-acid molecule with a molecular weight of 30.394 kDa.

### Surface-Enhanced Raman Spectroscopy of the *C. acnes* RT5 Biofilm Matrix

**FIGURE 3:**
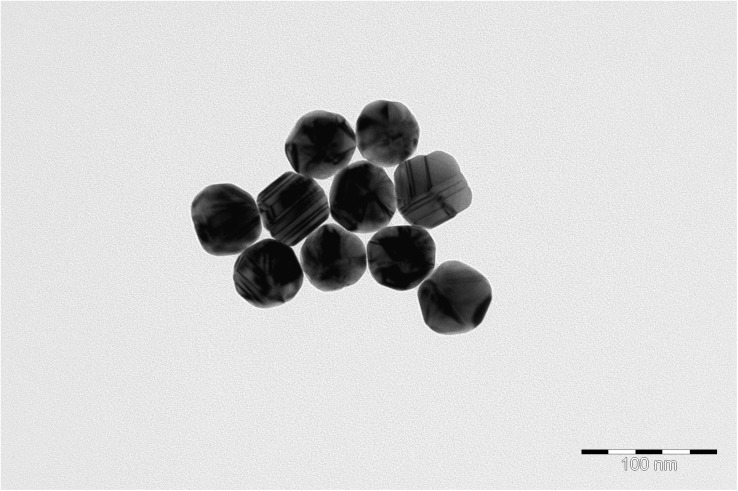
Transmission electron microscopy (TEM) image of nanoparticles.

**FIGURE 4:**
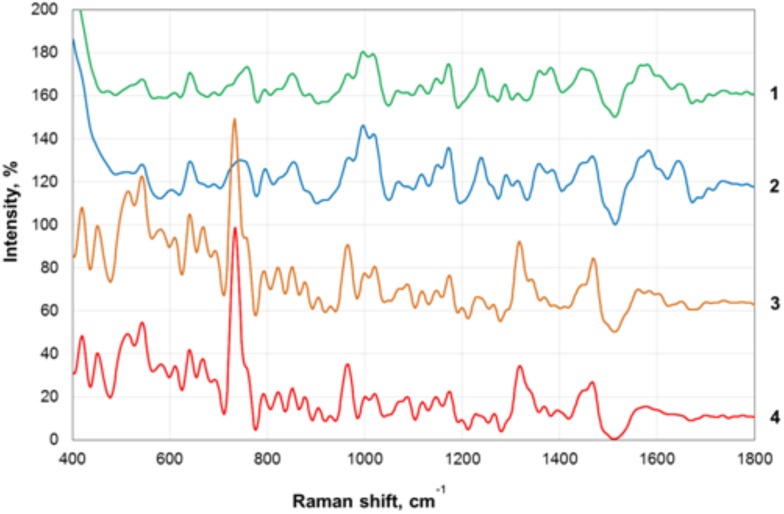
Surface-enhanced Raman scattering (SERS) spectra of the biofilm biomass and biofilm matrix of *C. acnes* RT5. 1—Upper phase of the matrix; 2—lower phase of the matrix; 3—biomass after matrix removal; 4—biomass of untreated biofilms.

Transmission electron microscopy images of nanoparticles are shown on Figure 3. Analysis of the SERS spectra of the isolated matrix in comparison with the biomass before and after matrix isolation showed more than 40 major peaks (Figure 4).

The registered SERS spectra of *C. acnes* biofilms and *C. acnes* cells separated from the matrix showed several peaks (735, 964, and 1,320 cm^−1^) that are characteristic of the spectra of many microorganisms (Kopitsyn et al., 2019). Major peaks in the SERS spectra of bacterial cells correspond to the oscillations of functional groups within redox complexes, such as NAD and flavin adenine dinucleotide (FAD). In particular, the 735-cm^−1^ peak corresponds to the stretching of the adenine cycle and that of the 964-cm^−1^ peak to swing/stretching along the N–C bond in adenine and guanine, and the 1,320-cm^−1^ peak is observed in NAD^+^ adsorbed on the silver electrode (Chen et al., 2002; Kairyte et al., 2012). These peaks are characteristic of microbial cells and were absent from the spectra of the separated matrix.

We plotted the spectrum of each sample on a graph with a displacement along the ordinate axis to avoid superposition of the spectra. Differences between the spectra of the isolated matrix and biofilm biomass suggest successful extraction of the matrix and not a suspension of cell debris after the extraction procedures. We show that SERS can be used to rapidly verify the successful extraction of the matrix. In addition, we obtained the total biomass spectra of the biofilm and the matrix, which will be added to the database of microorganism spectra and used to identify *C. acnes* in complex biological materials and samples with the help of SERS. SERS has the advantages of very simple sample preparation and rapid obtention of the results. It is, thus, a promising tool for bacterial detection. SERS spectroscopy by itself is also a potentially powerful tool for the detection of compounds that are generally normally present in bacterial biofilm matrices. However, a more complete picture, of course, needs to be built using a combination of SERS with other methods. To date, the weak point of SERS is the absence of a large spectra database of organic polymers, which greatly complicates the analysis. The essential value of the present data is as a contribution to the formation of this first SERS data base of bacterial spectra.

### Analysis of Polysaccharides

We analyzed both matrix and cell wall carbohydrates to compare them and generate as complete a picture as possible. We started first with cell wall sugars. The polysaccharide was isolated from disintegrated bacterial cells by stepwise extraction with 10% CCl_3_CO_2_H, first at 4°C for 48 h and then at 100°C for 5 min. The cold and hot extracts were separately dialyzed, lyophilized, and purified by gel permeation chromatography (GPC) on TSK HW-40 (S). Sugar analysis by GLC of the alditol acetates revealed a similar composition of polysaccharides in both extracts, which contained glucose, galactose, mannose, and GalNAc. The D configuration of glucose was determined by GLC of the acetylated (*S*)-2-octyl glycosides (Leontein and Lönngren, 1993). The D configuration of the other constituent monosaccharides was determined using known regularities in glycosylation effects on ^13^C NMR chemical shifts (Shashkov et al., 1988), as summarized and calculated by the GODDESS NMR simulation service (Kapaev and Toukach, 2015).

The ^1^H and ^13^C NMR spectra of both extracts were identical and showed signals of different intensities, indicating structural heterogeneity. The extracts were combined and studied by one-dimensional (1D) and 2D NMR spectroscopy.

The major series in the ^13^C NMR spectrum of the polysaccharide contained signals for five anomeric carbons at δ 99.8–102.7, three HOCH_2_-C groups (C-6 of Glc, Gal, and GalN) at δ 62.5–69.3, five nitrogen-bearing carbons (C-2 and C-3 of 2 ManN_3_NA and C-2 of GalN) at δ 52.0–54.5, and other sugar ring carbons in the region δ 68.2–82.3, and *N*-acetyl groups (CH_3_ at δ 23.4, CO at δ 175.4–176.4). These data are consistent with the composition of the PS determined by sugar analysis. The absence of signals from the region of δ 83–88, characteristic of furanosides (Bock and Pedersen, 1983; Lipkind et al., 1988), shows that all monosaccharide residues are in the pyranosidic form. The major series in the ^1^H NMR spectrum of the polysaccharide contained signals for five anomeric protons at δ 4.64–5.14, and other sugar protons in the region δ 3.35–4.41, and *N*-acetyl groups at δ 1.97–2.07.

**FIGURE 5:**
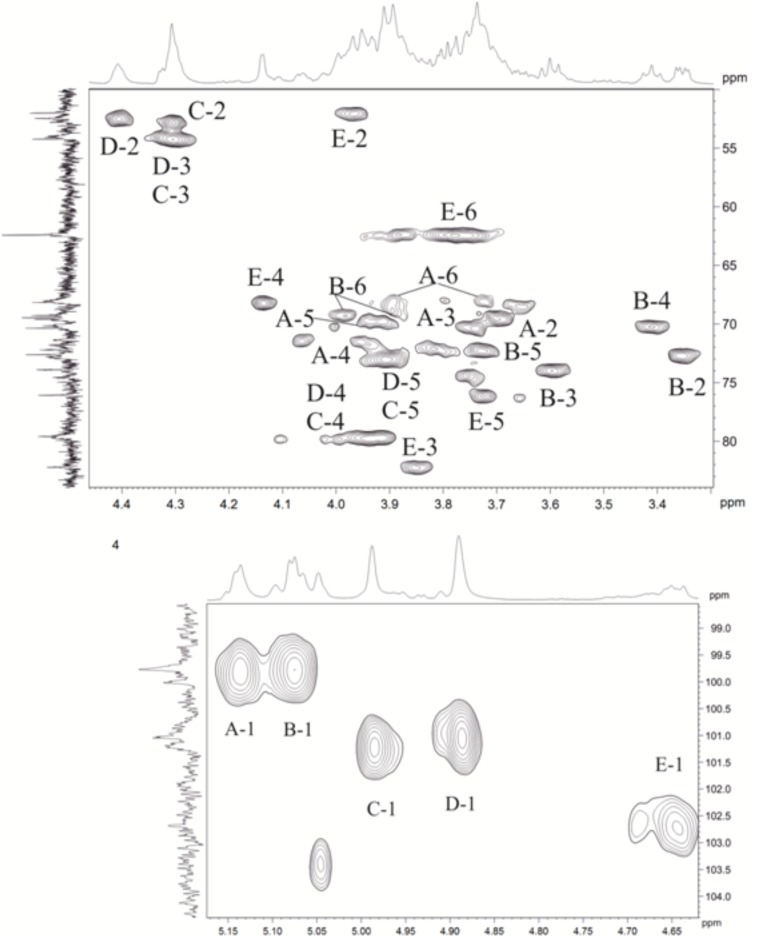
Parts of a 2D, ^1^H,^13^C multiplicity edited heteronuclear single quantum correlation (edHSQC) spectrum of the polysaccharide. The corresponding parts of the ^1^H and ^13^C nuclear magnetic resonance (NMR) spectra are shown along the horizontal and vertical axes, respectively. Numbers refer to protons, and the carbons of sugar units are designated by letters, as shown in Table 4.

The major series in the ^1^H and ^13^C NMR spectra of the polysaccharide were assigned following 2D ^1^H,^1^H COSY, ^1^H,^1^H TOCSY, ^1^H,^1^H ROESY, ^1^H,^13^C HSQC, and ^1^H,^13^C HMBC experiments (Table 4 and Figure 5), and spin systems for five residues each of Gal (**A**), Glc (**B**), ManNAc3NAcA (**C**), ManNAc3NAcA (**D**), and GalNAc (**E**) were identified. The assignment was based on correlations of H-1 and H-2 to H-6 for Glc, H-1 to H-2, and H-3 to H-5 for ManNAc3NAcA, and H-1 to H-4 for Gal and GalNAc in the TOCSY spectrum. The assignment within each spin system was performed using COSY, and relative configurations of the monosaccharides were determined based on ^3^*J*_H,H_ coupling constants. The H-6 signals for ManNAcA residues were found by H-5/C-6 correlations in the HMBC spectrum.

Relatively low-field positions of the C-5 signals at δ 72.9–76.2 in the 13C-NMR spectrum of the polysaccharide, relative to published data for the corresponding monosaccharides (Ranzinger et al., 2015; Toukach and Egorova, 2015), showed that ManNAc3NAcA (**C**), ManNAc3NAcA (**D**), and GalNAc (**E**) are β-linked. Similarly, the α configuration of Gal (**A**) and Glc (**B**) was inferred by relatively high-field positions of the C-5 signals at δ 71.5–72.3.

**FIGURE 6:**
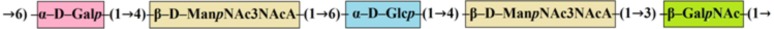
Structure of the polysaccharide of the matrix and cell wall of *C. acnes* HA043PA2.

Relatively low-field positions of the signals for C-6 of Gal (**A**), C-6 of Glc (**B**), C-4 of ManNAc3NAcA (**C**, **D**), and C-4 of GalNAc (**E**), relative to their positions in the corresponding non-substituted monosaccharides (Ranzinger et al., 2015; Toukach and Egorova, 2015), defined the positions of substitution of the monosaccharides in the polysaccharide. These data revealed the structure of the polysaccharide (Figure 6).

According to a systematic search in the Carbohydrate Structure Database (Toukach and Egorova, 2016), this structure (CSDB ID 29100) was reported earlier for *P. acnes* (equivalent of *C. acnes*) strain C7 (Nagaoka et al., 1985). In the matrix, it has a molecular weight of at least 12 kDa (because of pore size).

Analysis of the matrix polysaccharides showed that the dominant polysaccharide in the matrix is exactly the same as that of the *C. acnes* cell wall. Moreover, we found no signal of PNAG. Instead, we found *N*-acetylgalactosamine and *N*-acetylmannosamine. We did not find residues of 2-acetamido-2-deoxy-galactose in the polysaccharide, which is characteristic of PNAG. Instead we found residues of 2-acetamido-2-deoxy-galactose and 2,3-di-acetamido-2,3-dideoxy-mannuronic acid.

The signals from minor series were not assigned due to their low intensity.

## Discussion

Extracellular matrix is an essential component of microbial biofilms, performing a number of functions and playing a key role in the establishment and organization of biofilms. Biofilms are difficult to eradicate because of the matrix, which acts as a diffusion barrier for exogenous chemicals and biocides (Zhurina et al., 2014). Understanding the composition of the extracellular matrix may be crucial for predicting the properties of biofilms. This is essential both for fundamental science and applied areas (biotechnology, pharmacology, and medicine). For biotechnology, the physicochemical properties of the matrix, based on its biochemical composition, can be used to develop materials for biofilm carriers to obtain optimal biofilm growth. For example, this could be employed for wastewater treatment systems, which use anammox processes and require a substantial amount of anammox consortia biofilm biomass for optimal processing (Nozhevnikova et al., 2015). In medicine, understanding the matrix composition could also be crucial for the targeting of pathogens in chronic infections (Nozhevnikova et al., 2015).

Here, we carried out an extensive and comprehensive study of the *C. acnes* RT5 matrix. First, we produced fully mature 7-day biofilms (Tyner and Patel, 2016) on the surface of filters on a solid medium, which eliminated the material of the nutrient medium and made it possible to isolate the true biofilm matrix. We did not work with liquid media, as in previous studies (Jahns et al., 2016; Okuda et al., 2018). This technique allowed us to obtain pure biofilms without any planktonic cells (Plakunov et al., 2016). Second, in contrast to previous studies, we were not obliged to treat the biofilm with dispersin B and DNase, which automatically makes it impossible to detect a large subset of the matrix components. Third, the technique presented herein makes it possible to avoid any chemical alteration of the biofilm matrix before analysis. Thus, for the first time, we had access to the true relative quantitative ratio of the main components of the *C. acnes* matrix.

Most of the organic carbon (62.6%) in the matrix of *C. acnes* RT5 belonged to the carbohydrate component. The second most abundant components were peptides (9.6%), followed by DNA, accounting for approximately 4.0% of the total organic matter of the matrix. The remaining organic matter presumably contained metabolites and their intermediates, most involved in porphyrin synthesis. Analysis of the proteome of the biofilm showed that it contained more than 400 proteins. Chaperonin GroL was the most abundant protein and may participate in the organization of the matrix structure, due to its potential ability to bind to DNA of the matrix, based on the same annotated ability of its homolog Hspd1. The abundant hypothetical protein HMPREF9571_00996 is also interesting, because it has no conservative domains. According to BLAST pairwise alignments analysis, it is close to adhesins of *C. acnes*. So, it may play an important role in biofilm establishment and cell adhesion^4^. The highly abundant bacterial protein EF-Tu is a moonlighting protein; aside from its “classical” role in translation, it is involved in cytoskeletal maintenance (Soufo et al., 2010) and acts as a chaperone (Richarme, 1998). In Gram-positive bacteria, EF-Tu can also act as a sensor of human signal molecules (Mijouin et al., 2013). Thus, EF-Tu may play an important role in the matrix. The presence of a high amount of glycolysis enzymes in the matrix may be due to their abundance in cells and the normal cell lysis that occurs in biofilms, but this needs to be confirmed by future studies.

Enzymes of the extracted matrix were highly diverse, suggesting a high catalytic potential of the *C. acnes* matrix. For example, several dozen hydrolases, which are specific for different substrates, probably allow *C. acnes* biofilms to dissolve polymers of surrounding tissues of the host organism, in particular, human skin, which is responsible for their pathogenicity and probably determines the role of *C. acnes* in the development of acne and other diseases. Interestingly, we found peroxiredoxin, SOD, and catalase in the matrix. Such molecules are essential for an anaerobic bacterium, such as *C. acnes*, and could theoretically increase the resistance of *C. acnes* biofilms to unfavorable environmental factors. In addition, these molecules can have a positive antioxidant effect on skin. The potential antioxidant function can be performed by the novel protein of *C. acnes*—RoxP (Allhorn et al., 2016). RoxP was found to be highly secreted by some groups of *C. acnes*, especially the I group of former *P. acnes* strains (Andersson et al., 2019). We did not find RoxP in the matrix of *C. acnes* RT5 biofilms, which may be the result of our cultivation conditions. We did not expose the biomass of *C. acnes* to an aerobic atmosphere during formation of the biofilms, as did the authors. Second, it may reflect strain specificity, even inside the IA group of former *P. acnes*, of which *C. acnes* RT5 is a member (Fitz-Gibbon et al., 2013). Concerning other proteins, we do not know in what form ribosomes occur in the matrix: whole particles, separate subunits, protein aggregates, or separate proteins. Moreover, we detected only 43 of the more than 50 ribosomal proteins. It is possible that ribosomes are present in the matrix in all states. However, their potential role and the role of their proteins in the matrix of biofilms are still unclear.

We first carried out a complex analysis of the polysaccharides of the *C. acnes* RT5 biofilm matrix by NMR. The essential aim was not only to analyze the composition and structure of matrix exopolysaccharides but also to compare them with those of the cell wall. We found that the major matrix polysaccharide of *C. acnes* RT5 is the same as that of the cell wall, consisting of neutral and amino sugars (Ranzinger et al., 2015). This is essential, because it is the potential base of *C. acnes* biofilm formation and perhaps the essential material of the matrix. Okuda et al. (2018) recently reported that PNAG is a characteristic component of the biofilm matrix of various *C. acnes* strains. The authors performed dot-blot analysis using wheat germ agglutinin conjugated to horseradish peroxidase. They found PNAG in all the strains tested, whereas we did not find any PNAG residues in the present study. The different methodologies and strains used could explain the difference between these results. For example, the difference may be based on strain and environmental specificity. Different strains of *C. acnes* can produce different sugars in different amounts. There are currently no published data about any other polysaccharides in the biofilm matrix of *C. acnes*, but this possibility is reasonable. In addition, this hypothesis is consistent with the data of Holmberg et al. (2009), who showed differences in the ability to form biofilms between *C. acnes* strains from infected implants and those from skin. The synthesis of PNAG may be associated with a specific niche of biofilm growth and pathogenic processes; on the surface of implants, *C. acnes* starts to produce PNAG instead of a mannose-rich polymer. The difference can also be explained on the basis of minor sugar residues that we did not analyze. As Okuda et al. (2018) showed differences in PNAG production among seven strains, it is possible that our strain RT5 is not able to produce PNAG in high amounts. The polysaccharide material of the matrix was highly heterogeneous and difficult to analyze. It is possible that PNAG in the RT5 strain matrix is present in the undetected minor residues of matrix sugars. Finally, it is possible that the cells differ when they express the biofilm *versus* the normal planktonic phenotype. Thus, PNAG may be the result of synthesis in planktonic cells but not biofilms.

The essential element for biofilm matrix analysis is to determine the molecules that are a component of the normal planktonic cell envelope and those that are a result of the biofilm phenotype and form the matrix when a biofilm grows. Capsular polysaccharides can be distinguished as a separate group, and their participation in biofilm matrix formation is now the subject of discussion and studies (Limoli et al., 2015). In addition, capsular polysaccharides are usually associated with the cell envelope and are anchored there (Willis and Whitfield, 2013). Thus, because of the specificities of our method (physical disintegration of the biofilm), we studied secreted polymers (especially polysaccharides) that are synthesized during biofilm formation. In our case, the components of the matrix were the same as those reported by Nagaoka et al. (1985), in which they were described as cell wall polysaccharides. Two cell envelope lipoglycans (lipoarabinomannan and lipomannan) were described by Whale et al. (2004). Both also contain significant amounts of mannose, glucose, and galactose, but they were not shown to be extracellular. These polymers have a similar composition but are not the same (Whale et al., 2004). Because lipoglycans are macroamphiphilic and potentially have another function and origin than cell wall polysaccharides, cells in a biofilm matrix start to synthesize cell wall polysaccharides and not lipoglycans.

SERS has proven to be a simple and robust approach for biofilm matrix detection. As previously noted, it requires minor sample preparation and data analysis is rapid (Efrima and Zeiri, 2009; Chao and Zhang, 2012; Kopitsyn et al., 2019). Here, we conducted the first SERS analysis of a *C. acnes* biofilm matrix. Analysis of the SERS peaks also confirmed the isolation of the biofilm matrix, as more than 40 peaks in the matrix and biomass of the cells were different. The most intense peaks of both phases of the matrix were found at 370–386 cm^−1^. However, the spectra of SERS alone do not carry information about the nature of most of the matrix substances, because there is currently no SERS database of organic compounds, in particular organic polymers. Therefore, SERS is used in combination with other techniques, such as MS and NMR. The identification of spectra peaks requires the obtention of spectra for each compound of the matrix. Also, SERS is a relatively poor tool for the identification of molecules at low concentration. Nevertheless, we have successfully performed the first typing of biofilms and *C. acnes* matrix using the SERS method. This approach could help to determine the presence of *C. acnes* in complex biological samples in the future. It could also be used in the construction of a microorganism SERS spectra database. This database could allow the rapid and simple identification of microbes without the need of molecular biology approaches, such as polymerase chain reaction or fluorescence *in situ* hybridization.

## Data Availability

The raw data supporting the conclusions of this manuscript will be made available by the authors, without undue reservation, to any qualified researcher.

## Author Contributions

AG was responsible for *C. acnes* biofilm cultivation, development of the matrix extraction method, cell destruction control studies, biochemical studies of the matrix, initial preparation of all samples for MS, SERS, and NMR analysis, the analysis of all the results, writing the draft of the article, and the coordination of all experiments in France and Russia. EZ was responsible for the preparation of the samples for NMR, assignment of the NMR spectra, and writing a part of the article. AK extracted the polysaccharides, analyzed the sugars, and determined the absolute configuration of the monosaccharides. AS was responsible for recording of the 1D and 2D NMR spectra and assignment of the NMR spectra. YK helped in the data analysis and writing of the article. EB was responsible for the final preparation of the samples for SERS analysis and editing of the article. SM was responsible for the final preparation of the samples for the MS proteomics analysis. JH conducted the MS Orbitrap analysis and identified the proteins of the matrix. MG collected the SERS spectra. DK was responsible for the SERS data processing. MZ edited the final draft of the article and assisted in the cultivation of *C. acnes*. AN edited the article. VP and MF edited the article and were responsible for directing the work and development of the general strategy.

## Conflict of Interest Statement

The authors declare that the research was conducted in the absence of any commercial or financial relationships that could be construed as a potential conflict of interest.

## Footnotes

1 http://amigo.geneontology.org/amigo/term/GO:0001530

2 https://www.uniprot.org

3 https://www.ncbi.nlm.nih.gov

4 https://blast.ncbi.nlm.nih.gov/Blast.cgi
