# Supplementary material for: Composition of the Biofilm Matrix of Cutibacterium acnes Acneic Strain RT5
Source: Front Microbiol. 2019 Jun 21;10:1284. doi: 10.3389/fmicb.2019.01284 (PMC6598116; doi:10.3389/fmicb.2019.01284)
Supplement: Supplementary file 1 [file Table_1.docx]

Supplementary Material

1. Table S1. Proteins identified in the matrix of *C. acnes* RT5 biofilms. The proteins isolated only in the lower phase of the matrix are labeled red, and the proteins in the upper are in blue.

| **Accession** | **Description** | **MW [kDa]** | **Coverage** | **# AAs** |
| --- | --- | --- | --- | --- |
| 1. 327443705 | 1. bacterial NAD-glutamate dehydrogenase [*Cutibacterium acnes* HL043PA2] | 1. 172,238 | 1. 3,82 | 1. 1569 |
| 1. 327446964 | 1. DNA-directed RNA polymerase, beta' subunit [*Cutibacterium acnes* HL043PA2] | 1. 143,329 | 1. 10,67 | 1. 1293 |
| 1. 327445677 | 1. oxoglutarate dehydrogenase (succinyl-transferring), E1 component [*Cutibacterium acnes* HL043PA2] | 1. 136,695 | 1. 3,24 | 1. 1236 |
| 1. 327450411 | 1. pyruvate synthase [*Cutibacterium acnes* HL043PA2] | 1. 131,812 | 1. 12,13 | 1. 1204 |
| 1. 327446963 | 1. DNA-directed RNA polymerase, beta subunit [*Cutibacterium acnes* HL043PA2] | 1. 131,21 | 1. 7,17 | 1. 1185 |
| 1. 327445932 | 1. ribonucleoside-diphosphate reductase, adenosylcobalamin-dependent [*Cutibacterium acnes* HL043PA2] | 1. 103,873 | 1. 8,84 | 1. 950 |
| 1. 327443701 | 1. preprotein translocase, SecA subunit [*Cutibacterium acnes* HL043PA2] | 1. 101,327 | 1. 2,9 | 1. 898 |
| 1. 327445767 | 1. alanine--tRNA ligase [*Cutibacterium acnes* HL043PA2] | 1. 95,7476 | 1. 5,49 | 1. 892 |
| 1. 327445892 | 1. aconitate hydratase 1 [*Cutibacterium acnes* HL043PA2] | 1. 96,3455 | 1. 3,49 | 1. 888 |
| 1. 327447637 | 1. pyruvate, phosphate dikinase [*Cutibacterium acnes* HL043PA2] | 1. 96,4148 | 1. 25,93 | 1. 883 |
| 1. 327444294 | 1. membrane alanyl aminopeptidase [*Cutibacterium acnes* HL043PA2] | 1. 96,6945 | 1. 4,25 | 1. 870 |
| 1. 327444263 | 1. Anticodon-binding domain protein [*Cutibacterium acnes* HL043PA2] | 1. 97,2608 | 1. 1,84 | 1. 870 |
| 1. 327449698 | 1. ATPase family associated with various cellular activities (AAA) [*Cutibacterium acnes* HL043PA2] | 1. 93,2526 | 1. 2,73 | 1. 844 |
| 1. 327444364 | 1. phenylalanine--tRNA ligase, beta subunit [*Cutibacterium acnes* HL043PA2] | 1. 89,1879 | 1. 2,98 | 1. 839 |
| 1. 327446071 | 1. leucine--tRNA ligase [*Cutibacterium acnes* HL043PA2] | 1. 92,3907 | 1. 5,45 | 1. 826 |
| 1. 327442870 | 1. NADH dehydrogenase (quinone), G subunit [*Cutibacterium acnes* HL043PA2] | 1. 86,5539 | 1. 3,33 | 1. 812 |
| 1. 327446134 | 1. S1 RNA binding domain protein [*Cutibacterium acnes* HL043PA2] | 1. 88,6422 | 1. 3,11 | 1. 803 |
| 1. 327444160 | 1. guanosine pentaphosphate synthetase I/polyribonucleotide nucleotidyltransferase [*Cutibacterium acnes* HL043PA2] | 1. 78,8514 | 1. 13,64 | 1. 733 |
| 1. 327445140 | 1. methylmalonyl-CoA mutase large subunit [*Cutibacterium acnes* HL043PA2] | 1. 80,1163 | 1. 22,22 | 1. 729 |
| 1. 327446014 | 1. succinate dehydrogenase or fumarate reductase, flavoprotein subunit [*Cutibacterium acnes* HL043PA2] | 1. 78,0746 | 1. 5,35 | 1. 710 |
| 1. 327444579 | 1. translation elongation factor G [*Cutibacterium acnes* HL043PA2] | 1. 76,5564 | 1. 9,47 | 1. 697 |
| 1. 327445877 | 1. threonine--tRNA ligase [*Cutibacterium acnes* HL043PA2] | 1. 77,2639 | 1. 6,82 | 1. 689 |
| 1. 327444113 | 1. succinate dehydrogenase or fumarate reductase, flavoprotein subunit [*Cutibacterium acnes* HL043PA2] | 1. 75,7144 | 1. 12,59 | 1. 675 |
| 1. 327446943 | 1. 2-oxoacid:acceptor oxidoreductase, alpha subunit [*Cutibacterium acnes* HL043PA2] | 1. 70,328 | 1. 7,18 | 1. 655 |
| 1. 327449725 | 1. ferrochelatase [*Cutibacterium acnes* HL043PA2] | 1. 70,614 | 1. 11,54 | 1. 650 |
| 1. 327445141 | 1. methylmalonyl-CoA mutase, small subunit [*Cutibacterium acnes* HL043PA2] | 1. 69,0733 | 1. 32,7 | 1. 636 |
| 1. 327444300 | 1. AMP-binding enzyme [*Cutibacterium acnes* HL043PA2] | 1. 69,5205 | 1. 5,83 | 1. 635 |
| 1. 327448387 | 1. GTP-binding protein TypA [*Cutibacterium acnes* HL043PA2] | 1. 68,4269 | 1. 5,48 | 1. 620 |
| 1. 327447627 | 1. chaperone protein DnaK [*Cutibacterium acnes* HL043PA2] | 1. 66,3225 | 1. 41,49 | 1. 617 |
| 1. 327449464 | 1. glutamine-fructose-6-phosphate transaminase (isomerizing) [*Cutibacterium acnes* HL043PA2] | 1. 66,994 | 1. 8,13 | 1. 615 |
| 1. 327449171 | 1. AMP-binding enzyme [*Cutibacterium acnes* HL043PA2] | 1. 66,2291 | 1. 11,53 | 1. 607 |
| 1. 327445770 | 1. aspartate--tRNA ligase [*Cutibacterium acnes* HL043PA2] | 1. 66,4973 | 1. 18,98 | 1. 606 |
| 1. 327450412 | 1. pyridine nucleotide-disulfide oxidoreductase [*Cutibacterium acnes* HL043PA2] | 1. 66,4245 | 1. 12,23 | 1. 605 |
| 1. 327444157 | 1. thiamine pyrophosphate enzyme, N-terminal TPP binding domain protein [*Cutibacterium acnes* HL043PA2] | 1. 64,089 | 1. 13,9 | 1. 597 |
| 1. 327444184 | 1. translation initiation factor IF-2 [*Cutibacterium acnes* HL043PA2] | 1. 62,9378 | 1. 6 | 1. 583 |
| 1. 327447714 | 1. arginine--tRNA ligase [*Cutibacterium acnes* HL043PA2] | 1. 63,9594 | 1. 31,1 | 1. 582 |
| 1. 327444193 | 1. proline--tRNA ligase [*Cutibacterium acnes* HL043PA2] | 1. 63,6595 | 1. 32,36 | 1. 581 |
| 1. 327446077 | 1. malic enzyme, NAD binding domain protein [*Cutibacterium acnes* HL043PA2] | 1. 61,7616 | 1. 26,23 | 1. 568 |
| 1. 327446586 | 1. ABC transporter, ATP-binding protein [*Cutibacterium acnes* HL043PA2] | 1. 61,9042 | 1. 5,49 | 1. 565 |
| 1. 327443867 | 1. CTP synthase [*Cutibacterium acnes* HL043PA2] | 1. 60,6502 | 1. 8,01 | 1. 562 |
| 1. 327444155 | 1. hypothetical protein HMPREF9571_02286 [*Cutibacterium acnes* HL043PA2] | 1. 60,8078 | 1. 3,95 | 1. 557 |
| 1. 327449770 | 1. phosphoenolpyruvate-protein phosphotransferase [*Cutibacterium acnes* HL043PA2] | 1. 57,6929 | 1. 6,46 | 1. 557 |
| 1. 327449865 | 1. nicotinate-nucleotide--dimethylbenzimidazole phosphoribosyltransferase [*Cutibacterium acnes* HL043PA2] | 1. 58,6391 | 1. 5,58 | 1. 556 |
| 1. 327445697 | 1. ATP synthase F1, alpha subunit [*Cutibacterium acnes* HL043PA2] | 1. 58,872 | 1. 24,22 | 1. 545 |
| 1. 327449885 | 1. chaperonin GroL [*Cutibacterium acnes* HL043PA2] | 1. 56,8047 | 1. 44,3 | 1. 544 |
| 1. 327445845 | 1. phosphoglucomutase, alpha-D-glucose phosphate-specific [*Cutibacterium acnes* HL043PA2] | 1. 58,451 | 1. 7,55 | 1. 543 |
| 1. 327449490 | 1. chaperonin GroL [*Cutibacterium acnes* HL043PA2] | 1. 56,3943 | 1. 62,52 | 1. 531 |
| 1. 327444272 | 1. trigger factor [*Cutibacterium acnes* HL043PA2] | 1. 57,6801 | 1. 16,42 | 1. 530 |
| 1. 327444120 | 1. signal recognition particle protein [*Cutibacterium acnes* HL043PA2] | 1. 57,0852 | 1. 10,8 | 1. 528 |
| 1. 327448391 | 1. methylmalonyl-CoA carboxyltransferase 12S subunit [*Cutibacterium acnes* HL043PA2] | 1. 56,4265 | 1. 33,97 | 1. 524 |
| 1. 327447533 | 1. GMP synthase (glutamine-hydrolyzing) domain protein [*Cutibacterium acnes* HL043PA2] | 1. 55,5965 | 1. 10,64 | 1. 517 |
| 1. 327449145 | 1. phosphoribosylaminoimidazolecarboxamide formyltransferase/IMP cyclohydrolase [*Cutibacterium acnes* HL043PA2] | 1. 54,9575 | 1. 12,98 | 1. 516 |
| 1. 327446782 | 1. glycerol kinase [*Cutibacterium acnes* HL043PA2] | 1. 56,8473 | 1. 13,2 | 1. 515 |
| 1. 327445108 | 1. NAD(P)(+) transhydrogenase (AB-specific), alpha subunit [*Cutibacterium acnes* HL043PA2] | 1. 54,0842 | 1. 3,88 | 1. 515 |
| 1. 327450419 | 1. iron-sulfur cluster-binding protein [*Cutibacterium acnes* HL043PA2] | 1. 56,9632 | 1. 5,68 | 1. 511 |
| 1. 327445925 | 1. RNA polymerase sigma factor RpoD [*Cutibacterium acnes* HL043PA2] | 1. 55,6427 | 1. 3,72 | 1. 511 |
| 1. 327445032 | 1. cytosol aminopeptidase family, catalytic domain protein [*Cutibacterium acnes* HL043PA2] | 1. 52,0522 | 1. 25,98 | 1. 508 |
| 1. 327446016 | 1. glycine--tRNA ligase [*Cutibacterium acnes* HL043PA2] | 1. 57,1606 | 1. 11,66 | 1. 506 |
| 1. 327447643 | 1. succinate CoA transferase [*Cutibacterium acnes* HL043PA2] | 1. 55,0555 | 1. 15,28 | 1. 504 |
| 1. 327447528 | 1. inosine-5'-monophosphate dehydrogenase [*Cutibacterium acnes* HL043PA2] | 1. 53,6595 | 1. 24,01 | 1. 504 |
| 1. 327445824 | 1. aspartyl/glutamyl-tRNA(Asn/Gln) amidotransferase, A subunit [*Cutibacterium acnes* HL043PA2] | 1. 53,0335 | 1. 8,38 | 1. 501 |
| 1. 327447686 | 1. cellulase (glycosyl hydrolase family 5) [*Cutibacterium acnes* HL043PA2] | 1. 54,0388 | 1. 5,6 | 1. 500 |
| 1. 327446207 | 1. UDP-N-acetylmuramoyl-L-alanyl-D-glutamate--2,6-diaminopimelate ligase [*Cutibacterium acnes* HL043PA2] | 1. 52,2162 | 1. 5,21 | 1. 499 |
| 1. 327445083 | 1. aminopeptidase P domain protein [*Cutibacterium acnes* HL043PA2] | 1. 55,9833 | 1. 14,46 | 1. 498 |
| 1. 327446180 | 1. putative ribosomal protein S1 [*Cutibacterium acnes* HL043PA2] | 1. 55,1189 | 1. 25,1 | 1. 498 |
| 1. 327448392 | 1. conserved carboxylase domain protein [*Cutibacterium acnes* HL043PA2] | 1. 54,6555 | 1. 28,37 | 1. 497 |
| 1. 327445823 | 1. aspartyl/glutamyl-tRNA(Asn/Gln) amidotransferase, B subunit [*Cutibacterium acnes* HL043PA2] | 1. 54,2511 | 1. 9,46 | 1. 497 |
| 1. 327450434 | 1. lysine--tRNA ligase [*Cutibacterium acnes* HL043PA2] | 1. 55,2002 | 1. 4,66 | 1. 494 |
| 1. 327445089 | 1. cellulase (glycosyl hydrolase family 5) [*Cutibacterium acnes* HL043PA2] | 1. 53,0902 | 1. 5,07 | 1. 493 |
| 1. 327449790 | 1. glycine hydroxymethyltransferase [*Cutibacterium acnes* HL043PA2] | 1. 52,9026 | 1. 14,66 | 1. 491 |
| 1. 327445699 | 1. ATP synthase F1, beta subunit [*Cutibacterium acnes* HL043PA2] | 1. 53,4429 | 1. 40,49 | 1. 489 |
| 1. 327445715 | 1. putative UDP-N-acetylglucosamine 1-carboxyvinyltransferase [*Cutibacterium acnes* HL043PA2] | 1. 53,7019 | 1. 11,29 | 1. 487 |
| 1. 327446637 | 1. succinate-semialdehyde dehydrogenase [*Cutibacterium acnes* HL043PA2] | 1. 51,8836 | 1. 6,39 | 1. 485 |
| 1. 327446613 | 1. catalase [*Cutibacterium acnes* HL043PA2] | 1. 54,3092 | 1. 4,97 | 1. 483 |
| 1. 327444245 | 1. FeS assembly protein SufB [*Cutibacterium acnes* HL043PA2] | 1. 53,4615 | 1. 14,73 | 1. 482 |
| 1. 327448376 | 1. adenylosuccinate lyase [*Cutibacterium acnes* HL043PA2] | 1. 52,1836 | 1. 7,76 | 1. 477 |
| 1. 327445802 | 1. glycosyl hydrolase, family 1 [*Cutibacterium acnes* HL043PA2] | 1. 55,121 | 1. 3,57 | 1. 476 |
| 1. 327451109 | 1. argininosuccinate synthase [*Cutibacterium acnes* HL043PA2] | 1. 51,8299 | 1. 14,14 | 1. 474 |
| 1. 327445058 | 1. glutamine synthetase, type I [*Cutibacterium acnes* HL043PA2] | 1. 53,2083 | 1. 17,76 | 1. 473 |
| 1. 327449394 | 1. glutamate--tRNA ligase [*Cutibacterium acnes* HL043PA2] | 1. 53,6473 | 1. 9,11 | 1. 472 |
| 1. 327446201 | 1. UDP-N-acetylmuramate--L-alanine ligase [*Cutibacterium acnes* HL043PA2] | 1. 49,4491 | 1. 15,71 | 1. 471 |
| 1. 327446797 | 1. fumarate hydratase, class II [*Cutibacterium acnes* HL043PA2] | 1. 50,7034 | 1. 12,18 | 1. 468 |
| 1. 327443916 | 1. argininosuccinate lyase [*Cutibacterium acnes* HL043PA2] | 1. 50,4988 | 1. 10,9 | 1. 468 |
| 1. 327446003 | 1. putative alpha,alpha-trehalose-phosphate synthase (UDP-forming) [*Cutibacterium acnes* HL043PA2] | 1. 52,8332 | 1. 11,78 | 1. 467 |
| 1. 327445713 | 1. dihydrolipoyl dehydrogenase [*Cutibacterium acnes* HL043PA2] | 1. 49,6976 | 1. 16,27 | 1. 467 |
| 1. 327449929 | 1. UTP--glucose-1-phosphate uridylyltransferase [*Cutibacterium acnes* HL043PA2] | 1. 51,1487 | 1. 13,55 | 1. 465 |
| 1. 327446545 | 1. pyridine nucleotide-disulfide oxidoreductase [*Cutibacterium acnes* HL043PA2] | 1. 49,6044 | 1. 7,39 | 1. 460 |
| 1. 327449174 | 1. pyridine nucleotide-disulfide oxidoreductase [*Cutibacterium acnes* HL043PA2] | 1. 48,0198 | 1. 4,58 | 1. 459 |
| 1. 327445033 | 1. 2-oxoglutarate dehydrogenase, E2 component, dihydrolipoamide succinyltransferase [*Cutibacterium acnes* HL043PA2] | 1. 47,5648 | 1. 29,19 | 1. 459 |
| 1. 327445885 | 1. putative tryptophan 2,3-dioxygenase [*Cutibacterium acnes* HL043PA2] | 1. 50,7493 | 1. 3,71 | 1. 458 |
| 1. 327446190 | 1. pyruvate kinase [*Cutibacterium acnes* HL043PA2] | 1. 49,7848 | 1. 33,62 | 1. 458 |
| 1. 327445732 | 1. ribosome biogenesis GTPase Der [*Cutibacterium acnes* HL043PA2] | 1. 49,7877 | 1. 5,26 | 1. 456 |
| 1. 327445776 | 1. histidine--tRNA ligase [*Cutibacterium acnes* HL043PA2] | 1. 50,2328 | 1. 9,03 | 1. 454 |
| 1. 327443723 | 1. putative hydrolase [*Cutibacterium acnes* HL043PA2] | 1. 49,9688 | 1. 4,42 | 1. 453 |
| 1. 327448394 | 1. PAS domain S-box protein [*Cutibacterium acnes* HL043PA2] | 1. 49,1598 | 1. 14,16 | 1. 452 |
| 1. 327449180 | 1. TQXA domain protein [*Cutibacterium acnes* HL043PA2] | 1. 47,897 | 1. 5,97 | 1. 452 |
| 1. 327446616 | 1. putative aspartate ammonia-lyase [*Cutibacterium acnes* HL043PA2] | 1. 49,1785 | 1. 21,51 | 1. 451 |
| 1. 327449458 | 1. phosphoglucosamine mutase [*Cutibacterium acnes* HL043PA2] | 1. 47,5134 | 1. 18,22 | 1. 450 |
| 1. 327450255 | 1. amine oxidase (flavin-containing) [*Cutibacterium acnes* HL043PA2] | 1. 47,5902 | 1. 8,02 | 1. 449 |
| 1. 327443351 | 1. peptidase dimerization domain protein [*Cutibacterium acnes* HL043PA2] | 1. 47,1711 | 1. 14,32 | 1. 447 |
| 1. 327449220 | 1. nicotinate phosphoribosyltransferase [*Cutibacterium acnes* HL043PA2] | 1. 47,3962 | 1. 13,9 | 1. 446 |
| 1. 327449724 | 1. glutamyl-tRNAGlu reductase domain protein [*Cutibacterium acnes* HL043PA2] | 1. 47,105 | 1. 17,04 | 1. 446 |
| 1. 327445849 | 1. hypothetical protein HMPREF9571_01585 [*Cutibacterium acnes* HL043PA2] | 1. 48,4552 | 1. 29,66 | 1. 445 |
| 1. 327444207 | 1. Glu/Leu/Phe/Val dehydrogenase, dimerization domain protein [*Cutibacterium acnes* HL043PA2] | 1. 48,1119 | 1. 18,65 | 1. 445 |
| 1. 327445963 | 1. amidohydrolase family protein [*Cutibacterium acnes* HL043PA2] | 1. 47,6689 | 1. 6,29 | 1. 445 |
| 1. 327442869 | 1. NADH oxidoreductase (quinone), F subunit [*Cutibacterium acnes* HL043PA2] | 1. 48,5162 | 1. 11,71 | 1. 444 |
| 1. 327446834 | 1. putative YcaO-like family [*Cutibacterium acnes* HL043PA2] | 1. 48,3883 | 1. 12,44 | 1. 442 |
| 1. 327445680 | 1. homoserine dehydrogenase [*Cutibacterium acnes* HL043PA2] | 1. 46,6288 | 1. 14,29 | 1. 441 |
| 1. 327444166 | 1. glycine betaine/L-proline transport ATP binding subunit [*Cutibacterium acnes* HL043PA2] | 1. 48,225 | 1. 13,21 | 1. 439 |
| 1. 327449142 | 1. 3,4-dihydroxy-2-butanone-4-phosphate synthase [*Cutibacterium acnes* HL043PA2] | 1. 46,6248 | 1. 16,67 | 1. 438 |
| 1. 327449718 | 1. glutamate-1-semialdehyde-2,1-aminomutase [*Cutibacterium acnes* HL043PA2] | 1. 45,4128 | 1. 16,51 | 1. 436 |
| 1. 327449935 | 1. MoeA N-terminal region (domain I and II) [*Cutibacterium acnes* HL043PA2] | 1. 45,5819 | 1. 6,9 | 1. 435 |
| 1. 327442867 | 1. NADH dehydrogenase subunit D [*Cutibacterium acnes* HL043PA2] | 1. 47,9817 | 1. 8,31 | 1. 433 |
| 1. 327445779 | 1. hypothetical protein HMPREF9571_01515 [*Cutibacterium acnes* HL043PA2] | 1. 49,0619 | 1. 20,37 | 1. 432 |
| 1. 327448378 | 1. adenylosuccinate synthase [*Cutibacterium acnes* HL043PA2] | 1. 47,33 | 1. 20,83 | 1. 432 |
| 1. 327446554 | 1. nucleotide sugar dehydrogenase [*Cutibacterium acnes* HL043PA2] | 1. 46,8528 | 1. 15,05 | 1. 432 |
| 1. 327444291 | 1. hypothetical protein HMPREF9571_02424 [*Cutibacterium acnes* HL043PA2] | 1. 47,0956 | 1. 5,13 | 1. 429 |
| 1. 327445165 | 1. aminopeptidase I zinc metalloprotease (M18) [*Cutibacterium acnes* HL043PA2] | 1. 45,9351 | 1. 5,14 | 1. 428 |
| 1. 327449197 | 1. phosphoribosylaminoimidazole carboxylase, ATPase subunit [*Cutibacterium acnes* HL043PA2] | 1. 45,8699 | 1. 6,34 | 1. 426 |
| 1. 327449991 | 1. phosphopyruvate hydratase [*Cutibacterium acnes* HL043PA2] | 1. 45,514 | 1. 46,24 | 1. 426 |
| 1. 327449117 | 1. serine--tRNA ligase [*Cutibacterium acnes* HL043PA2] | 1. 46,5027 | 1. 9,43 | 1. 424 |
| 1. 327444244 | 1. FeS assembly protein SufD [*Cutibacterium acnes* HL043PA2] | 1. 45,8413 | 1. 17,22 | 1. 424 |
| 1. 327447713 | 1. hypothetical protein HMPREF9571_00996 [*Cutibacterium acnes* HL043PA2] | 1. 43,2017 | 1. 46,23 | 1. 424 |
| 1. 327449848 | 1. sirohydrochlorin cobaltochelatase [*Cutibacterium acnes* HL043PA2] | 1. 45,4854 | 1. 4,74 | 1. 422 |
| 1. 327448438 | 1. phosphoribosylaminoimidazolesuccinocarboxamide synthase [*Cutibacterium acnes* HL043PA2] | 1. 46,4004 | 1. 15,24 | 1. 420 |
| 1. 327446199 | 1. cell division protein FtsZ [*Cutibacterium acnes* HL043PA2] | 1. 42,8086 | 1. 14,87 | 1. 417 |
| 1. 327446603 | 1. periplasmic binding protein [*Cutibacterium acnes* HL043PA2] | 1. 45,0928 | 1. 7,45 | 1. 416 |
| 1. 327446722 | 1. DNA polymerase III, beta subunit [*Cutibacterium acnes* HL043PA2] | 1. 44,0732 | 1. 16,18 | 1. 414 |
| 1. 327444241 | 1. cysteine desulfurase, SufS subfamily [*Cutibacterium acnes* HL043PA2] | 1. 44,4723 | 1. 8,23 | 1. 413 |
| 1. 327445752 | 1. phosphopantothenoylcysteine decarboxylase/phosphopantothenate--cysteine ligase [*Cutibacterium acnes* HL043PA2] | 1. 42,7904 | 1. 4,39 | 1. 410 |
| 1. 327445093 | 1. glucose-1-phosphate adenylyltransferase [*Cutibacterium acnes* HL043PA2] | 1. 44,7392 | 1. 5,64 | 1. 408 |
| 1. 7444204 | 1. 1-deoxy-D-xylulose 5-phosphate reductoisomerase [*Cutibacterium acnes* HL043PA2] | 1. 42,3727 | 1. 10,07 | 1. 407 |
| 1. 327445166 | 1. putative flagellar protein FliS [*Cutibacterium acnes* HL043PA2] | 1. 44,2509 | 1. 8,62 | 1. 406 |
| 1. 327446041 | 1. hypothetical protein HMPREF9571_01783 [*Cutibacterium acnes* HL043PA2] | 1. 43,1674 | 1. 23,95 | 1. 405 |
| 1. 327445862 | 1. Phosphofructokinase [*Cutibacterium acnes* HL043PA2] | 1. 43,4902 | 1. 32,43 | 1. 404 |
| 1. 327446143 | 1. phosphoglycerate kinase [*Cutibacterium acnes* HL043PA2] | 1. 42,1912 | 1. 47,01 | 1. 402 |
| 1. 327449390 | 1. translation elongation factor Tu [*Cutibacterium acnes* HL043PA2] | 1. 44,1124 | 1. 37,78 | 1. 397 |
| 1. 327444122 | 1. signal recognition particle-docking protein FtsY [*Cutibacterium acnes* HL043PA2] | 1. 41,2367 | 1. 12,37 | 1. 396 |
| 1. 327445985 | 1. ATPase family associated with various cellular activities (AAA) [*Cutibacterium acnes* HL043PA2] | 1. 42,687 | 1. 13,16 | 1. 395 |
| 1. 327449826 | 1. glycine C-acetyltransferase [*Cutibacterium acnes* HL043PA2] | 1. 42,8816 | 1. 14,72 | 1. 394 |
| 1. 327446048 | 1. putative chaperone protein DnaJ [*Cutibacterium acnes* HL043PA2] | 1. 41,8327 | 1. 12,76 | 1. 392 |
| 1. 327449137 | 1. succinate-CoA ligase, beta subunit [*Cutibacterium acnes* HL043PA2] | 1. 41,3311 | 1. 37,85 | 1. 391 |
| 1. 327447636 | 1. FHA domain protein [*Cutibacterium acnes* HL043PA2] | 1. 41,1582 | 1. 8,95 | 1. 391 |
| 1. 327451178 | 1. TIGR00730 family protein [*Cutibacterium acnes* HL043PA2] | 1. 42,206 | 1. 4,11 | 1. 389 |
| 1. 327443362 | 1. NlpC/P60 family protein [*Cutibacterium acnes* HL043PA2] | 1. 40,3889 | 1. 24,48 | 1. 388 |
| 1. 327444119 | 1. amidohydrolase family protein [*Cutibacterium acnes* HL043PA2] | 1. 41,9412 | 1. 8,27 | 1. 387 |
| 1. 327445088 | 1. hypothetical protein HMPREF9571_02074 [*Cutibacterium acnes* HL043PA2] | 1. 40,7534 | 1. 8,55 | 1. 386 |
| 1. 327443707 | 1. tryptophan--tRNA ligase [*Cutibacterium acnes* HL043PA2] | 1. 41,4214 | 1. 6,77 | 1. 384 |
| 1. 327449759 | 1. phosphate ABC transporter, phosphate-binding protein PstS [*Cutibacterium acnes* HL043PA2] | 1. 39,5228 | 1. 31,33 | 1. 383 |
| 1. 327449121 | 1. acyl-CoA dehydrogenase, C-terminal domain protein [*Cutibacterium acnes* HL043PA2] | 1. 41,2717 | 1. 5,24 | 1. 382 |
| 1. 327448424 | 1. chaperone protein DnaJ [*Cutibacterium acnes* HL043PA2] | 1. 40,5873 | 1. 8,16 | 1. 380 |
| 1. 327445154 | 1. Amidinotransferase [*Cutibacterium acnes* HL043PA2] | 1. 41,6477 | 1. 8,73 | 1. 378 |
| 1. 327445101 | 1. Amidinotransferase [*Cutibacterium acnes* HL043PA2] | 1. 43,329 | 1. 28,99 | 1. 376 |
| 1. 327446955 | 1. UDP-N-acetylenolpyruvoylglucosamine reductase domain protein [*Cutibacterium acnes* HL043PA2] | 1. 39,863 | 1. 5,59 | 1. 376 |
| 1. 327449922 | 1. putative N-acetylglucosamine-6-phosphate deacetylase [*Cutibacterium acnes* HL043PA2] | 1. 39,1119 | 1. 10,64 | 1. 376 |
| 1. 327445144 | 1. nucleotide sugar dehydrogenase [*Cutibacterium acnes* HL043PA2] | 1. 41,4731 | 1. 6,12 | 1. 376 |
| 1. 327446202 | 1. putative undecaprenyldiphospho-muramoylpentapeptide beta-N-acetylglucosaminyltransferase [*Cutibacterium acnes* HL043PA2] | 1. 38,2132 | 1. 6,72 | 1. 372 |
| 1. 327443908 | 1. peptide chain release factor 2 [*Cutibacterium acnes* HL043PA2] | 1. 41,4429 | 1. 17,25 | 1. 371 |
| 1. 327446217 | 1. aminomethyltransferase [*Cutibacterium acnes* HL043PA2] | 1. 39,546 | 1. 4,85 | 1. 371 |
| 1. 327445351 | 1. hypothetical protein HMPREF9571_01989 [*Cutibacterium acnes* HL043PA2] | 1. 39,0608 | 1. 39,08 | 1. 371 |
| 1. 327446565 | 1. DegT/DnrJ/EryC1/StrS aminotransferase family protein [*Cutibacterium acnes* HL043PA2] | 1. 39,2338 | 1. 21,35 | 1. 370 |
| 1. 327444578 | 1. alanine dehydrogenase [*Cutibacterium acnes* HL043PA2] | 1. 38,1094 | 1. 14,05 | 1. 370 |
| 1. 327444365 | 1. phenylalanine--tRNA ligase, alpha subunit [*Cutibacterium acnes* HL043PA2] | 1. 40,1231 | 1. 5,96 | 1. 369 |
| 1. 327444248 | 1. ABC transporter, solute-binding protein [*Cutibacterium acnes* HL043PA2] | 1. 39,7604 | 1. 5,98 | 1. 368 |
| 1. 327447529 | 1. IMP dehydrogenase family protein [*Cutibacterium acnes* HL043PA2] | 1. 39,3409 | 1. 40,87 | 1. 367 |
| 1. 327445105 | 1. succinyldiaminopimelate transaminase [*Cutibacterium acnes* HL043PA2] | 1. 39,0519 | 1. 4,9 | 1. 367 |
| 1. 327449727 | 1. hypothetical protein HMPREF9571_00293 [*Cutibacterium acnes* HL043PA2] | 1. 40,2009 | 1. 10,11 | 1. 366 |
| 1. 327449816 | 1. ABC transporter, ATP-binding protein [*Cutibacterium acnes* HL043PA2] | 1. 39,6844 | 1. 9,84 | 1. 366 |
| 1. 327447700 | 1. oxidoreductase, FAD/FMN-binding protein [*Cutibacterium acnes* HL043PA2] | 1. 39,0556 | 1. 15,85 | 1. 366 |
| 1. 327443902 | 1. D-ala D-ala ligase N-terminal domain protein [*Cutibacterium acnes* HL043PA2] | 1. 39,2359 | 1. 5,21 | 1. 365 |
| 1. 327449152 | 1. malate dehydrogenase [*Cutibacterium acnes* HL043PA2] | 1. 38,7348 | 1. 33,52 | 1. 364 |
| 1. 327446729 | 1. FemAB family protein [*Cutibacterium acnes* HL043PA2] | 1. 40,7178 | 1. 9,64 | 1. 363 |
| 1. 327443892 | 1. branched-chain-amino-acid transaminase [*Cutibacterium acnes* HL043PA2] | 1. 39,8763 | 1. 31,96 | 1. 363 |
| 1. 327446194 | 1. DivIVA domain protein [*Cutibacterium acnes* HL043PA2] | 1. 40,1145 | 1. 38,5 | 1. 361 |
| 1. 327446942 | 1. thiamine pyrophosphate enzyme, C-terminal TPP binding domain protein [*Cutibacterium acnes* HL043PA2] | 1. 38,4973 | 1. 9,97 | 1. 361 |
| 1. 327445681 | 1. threonine synthase [*Cutibacterium acnes* HL043PA2] | 1. 37,4223 | 1. 6,15 | 1. 358 |
| 1. 327446550 | 1. UDP-N-acetylglucosamine 2-epimerase [*Cutibacterium acnes* HL043PA2] | 1. 38,4964 | 1. 7,28 | 1. 357 |
| 1. 327443873 | 1. peptidyl-prolyl cis-trans isomerase, FKBP-type [*Cutibacterium acnes* HL043PA2] | 1. 36,8285 | 1. 10,64 | 1. 357 |
| 1. 327445771 | 1. hypothetical protein HMPREF9571_01507 [*Cutibacterium acnes* HL043PA2] | 1. 40,1423 | 1. 48,31 | 1. 356 |
| 1. 327446649 | 1. UDP-glucose 4-epimerase [*Cutibacterium acnes* HL043PA2] | 1. 38,5445 | 1. 11,33 | 1. 353 |
| 1. 327447668 | 1. pyruvate dehydrogenase E1 component, alpha subunit [*Cutibacterium acnes* HL043PA2] | 1. 38,2386 | 1. 6,27 | 1. 351 |
| 1. 327445153 | 1. ornithine carbamoyltransferase [*Cutibacterium acnes* HL043PA2] | 1. 38,5804 | 1. 23,14 | 1. 350 |
| 1. 327446776 | 1. thioredoxin-disulfide reductase [*Cutibacterium acnes* HL043PA2] | 1. 37,2393 | 1. 10,57 | 1. 350 |
| 1. 327445138 | 1. LAO/AO transport system ATPase [*Cutibacterium acnes* HL043PA2] | 1. 37,2082 | 1. 19,83 | 1. 348 |
| 1. 327445947 | 1. RecA protein [*Cutibacterium acnes* HL043PA2] | 1. 37,1302 | 1. 36,21 | 1. 348 |
| 1. 327448421 | 1. GroES-like protein [*Cutibacterium acnes* HL043PA2] | 1. 36,9161 | 1. 8,07 | 1. 347 |
| 1. 327449734 | 1. aspartate-semialdehyde dehydrogenase [*Cutibacterium acnes* HL043PA2] | 1. 35,9806 | 1. 22,77 | 1. 347 |
| 1. 327449480 | 1. putative glycoprotease GCP [*Cutibacterium acnes* HL043PA2] | 1. 35,9716 | 1. 16,43 | 1. 347 |
| 1. 327446664 | 1. ABC transporter, ATP-binding protein [*Cutibacterium acnes* HL043PA2] | 1. 36,8586 | 1. 13,37 | 1. 344 |
| 1. 327446832 | 1. putative streptolysin associated protein SagB [*Cutibacterium acnes* HL043PA2] | 1. 36,8756 | 1. 20,7 | 1. 343 |
| 1. 327443887 | 1. ketol-acid reductoisomerase [*Cutibacterium acnes* HL043PA2] | 1. 37,3436 | 1. 6,43 | 1. 342 |
| 1. 327445684 | 1. putative transcription termination factor Rho [*Cutibacterium acnes* HL043PA2] | 1. 38,3643 | 1. 14,91 | 1. 342 |
| 1. 327446730 | 1. alanine racemase domain protein [*Cutibacterium acnes* HL043PA2] | 1. 37,1985 | 1. 5,56 | 1. 342 |
| 1. 327448397 | 1. fructose-bisphosphate aldolase, class II [*Cutibacterium acnes* HL043PA2] | 1. 36,8194 | 1. 27,27 | 1. 341 |
| 1. 327444163 | 1. riboflavin biosynthesis protein RibF [*Cutibacterium acnes* HL043PA2] | 1. 36,6851 | 1. 7,65 | 1. 340 |
| 1. 327446049 | 1. heat-inducible transcription repressor HrcA [*Cutibacterium acnes* HL043PA2] | 1. 36,3582 | 1. 9,14 | 1. 339 |
| 1. 327449434 | 1. DNA-directed RNA polymerase, alpha subunit [*Cutibacterium acnes* HL043PA2] | 1. 36,8267 | 1. 36,39 | 1. 338 |
| 1. 327449834 | 1. oxidoreductase, zinc-binding dehydrogenase family protein [*Cutibacterium acnes* HL043PA2] | 1. 36,165 | 1. 9,17 | 1. 338 |
| 1. 327450261 | 1. hypothetical protein HMPREF9571_00242 [*Cutibacterium acnes* HL043PA2] | 1. 36,2868 | 1. 5,04 | 1. 337 |
| 1. 327446033 | 1. prolyl aminopeptidase [*Cutibacterium acnes* HL043PA2] | 1. 37,7714 | 1. 17,01 | 1. 335 |
| 1. 327449699 | 1. hypothetical protein HMPREF9571_00265 [*Cutibacterium acnes* HL043PA2] | 1. 37,4598 | 1. 7,16 | 1. 335 |
| 1. 327446144 | 1. glyceraldehyde-3-phosphate dehydrogenase, type I [*Cutibacterium acnes* HL043PA2] | 1. 35,9276 | 1. 73,13 | 1. 335 |
| 1. 327451095 | 1. hypothetical protein HMPREF9571_00034, partial [*Cutibacterium acnes* HL043PA2] | 1. 34,3863 | 1. 34,63 | 1. 335 |
| 1. 327447667 | 1. Transketolase, pyridine binding domain protein [*Cutibacterium acnes* HL043PA2] | 1. 36,3015 | 1. 21,26 | 1. 334 |
| 1. 327443377 | 1. dihydroxyacetone kinase, DhaK subunit [*Cutibacterium acnes* HL043PA2] | 1. 34,6556 | 1. 10,21 | 1. 333 |
| 1. 327445977 | 1. beta-ketoacyl-acyl-carrier-protein synthase III [*Cutibacterium acnes* HL043PA2] | 1. 35,2539 | 1. 8,13 | 1. 332 |
| 1. 327449896 | 1. AP endonuclease, family 2 [*Cutibacterium acnes* HL043PA2] | 1. 36,5881 | 1. 14,46 | 1. 332 |
| 1. 327444359 | 1. tyrosine--tRNA ligase [*Cutibacterium acnes* HL043PA2] | 1. 36,7827 | 1. 5,44 | 1. 331 |
| 1. 327446564 | 1. oxidoreductase, NAD-binding domain protein [*Cutibacterium acnes* HL043PA2] | 1. 35,5402 | 1. 26,67 | 1. 330 |
| 1. 327446833 | 1. hypothetical protein HMPREF9571_01087 [*Cutibacterium acnes* HL043PA2] | 1. 35,7231 | 1. 10,06 | 1. 328 |
| 1. 327450410 | 1. putative dihydroorotate dehydrogenase 2 [*Cutibacterium acnes* HL043PA2] | 1. 34,6635 | 1. 32,42 | 1. 327 |
| 1. 327442879 | 1. polyprenyl synthetase [*Cutibacterium acnes* HL043PA2] | 1. 34,6627 | 1. 8,95 | 1. 324 |
| 1. 327444188 | 1. transcription termination factor NusA [*Cutibacterium acnes* HL043PA2] | 1. 35,7166 | 1. 4,64 | 1. 323 |
| 1. 327448382 | 1. dihydrodipicolinate synthetase family [*Cutibacterium acnes* HL043PA2] | 1. 34,4606 | 1. 6,81 | 1. 323 |
| 1. 327445148 | 1. oxidoreductase, aldo/keto reductase family protein [*Cutibacterium acnes* HL043PA2] | 1. 34,8244 | 1. 17,08 | 1. 322 |
| 1. 327446078 | 1. L-lactate dehydrogenase [*Cutibacterium acnes* HL043PA2] | 1. 33,9924 | 1. 6,83 | 1. 322 |
| 1. 327443371 | 1. lactate/malate dehydrogenase, NAD binding domain protein [*Cutibacterium acnes* HL043PA2] | 1. 33,6428 | 1. 15,89 | 1. 321 |
| 1. 327446213 | 1. ATPase family associated with various cellular activities (AAA) [*Cutibacterium acnes* HL043PA2] | 1. 34,395 | 1. 9,12 | 1. 318 |
| 1. 327445829 | 1. methionine synthase, vitamin-B12 independent [*Cutibacterium acnes* HL043PA2] | 1. 33,5973 | 1. 5,71 | 1. 315 |
| 1. 327446780 | 1. putative sugar-binding domain protein [*Cutibacterium acnes* HL043PA2] | 1. 33,5603 | 1. 6,98 | 1. 315 |
| 1. 327448409 | 1. putative fructose-bisphosphate aldolase class-I [*Cutibacterium acnes* HL043PA2] | 1. 34,724 | 1. 37,26 | 1. 314 |
| 1. 327445964 | 1. aspartate carbamoyltransferase [*Cutibacterium acnes* HL043PA2] | 1. 34,7149 | 1. 28,66 | 1. 314 |
| 1. 327445698 | 1. ATP synthase F1, gamma subunit [*Cutibacterium acnes* HL043PA2] | 1. 34,5417 | 1. 23,89 | 1. 314 |
| 1. 327445843 | 1. thioredoxin [*Cutibacterium acnes* HL043PA2] | 1. 33,1149 | 1. 5,43 | 1. 313 |
| 1. 327449719 | 1. porphobilinogen synthase [*Cutibacterium acnes* HL043PA2] | 1. 33,4195 | 1. 23,47 | 1. 311 |
| 1. 327446950 | 1. enoyl-CoA hydratase/isomerase family protein [*Cutibacterium acnes* HL043PA2] | 1. 33,1245 | 1. 7,72 | 1. 311 |
| 1. 327449976 | 1. ribose-phosphate diphosphokinase [*Cutibacterium acnes* HL043PA2] | 1. 33,9934 | 1. 9,35 | 1. 310 |
| 1. 327449994 | 1. Ppx/GppA phosphatase family protein [*Cutibacterium acnes* HL043PA2] | 1. 32,7367 | 1. 6,45 | 1. 310 |
| 1. 327445807 | 1. N-acetylmuramic acid 6-phosphate etherase [*Cutibacterium acnes* HL043PA2] | 1. 31,2779 | 1. 6,58 | 1. 304 |
| 1. 327446958 | 1. transcription termination/antitermination factor NusG [*Cutibacterium acnes* HL043PA2] | 1. 33,4752 | 1. 9,9 | 1. 303 |
| 1. 327445040 | 1. Tat pathway signal sequence domain protein [*Cutibacterium acnes* HL043PA2] | 1. 32,5854 | 1. 8,91 | 1. 303 |
| 1. 327449970 | 1. hydrolase, TatD family [*Cutibacterium acnes* HL043PA2] | 1. 32,3613 | 1. 8,94 | 1. 302 |
| 1. 327445739 | 1. hypothetical protein HMPREF9571_01475 [*Cutibacterium acnes* HL043PA2] | 1. 33,2098 | 1. 10,96 | 1. 301 |
| 1. 327446059 | 1. naphthoate synthase [*Cutibacterium acnes* HL043PA2] | 1. 33,1334 | 1. 8,31 | 1. 301 |
| 1. 327449401 | 1. 50S ribosomal protein L4 [*Cutibacterium acnes* HL043PA2] | 1. 32,2785 | 1. 43,52 | 1. 301 |
| 1. 327445152 | 1. carbamate kinase [*Cutibacterium acnes* HL043PA2] | 1. 31,9773 | 1. 8,31 | 1. 301 |
| 1. 327449477 | 1. hydrolase, P-loop family [*Cutibacterium acnes* HL043PA2] | 1. 30,7598 | 1. 9,09 | 1. 297 |
| 1. 327449138 | 1. succinate-CoA ligase, alpha subunit [*Cutibacterium acnes* HL043PA2] | 1. 30,5618 | 1. 48,15 | 1. 297 |
| 1. 327446684 | 1. putative histidinol-phosphate transaminase [*Cutibacterium acnes* HL043PA2] | 1. 32,0123 | 1. 13,22 | 1. 295 |
| 1. 327448374 | 1. putative alkyl hydroperoxide reductase, F subunit, partial [*Cutibacterium acnes* HL043PA2] | 1. 31,1971 | 1. 6,78 | 1. 295 |
| 1. 327445091 | 1. dihydrodipicolinate synthase [*Cutibacterium acnes* HL043PA2] | 1. 30,6707 | 1. 7,17 | 1. 293 |
| 1. 327451296 | 1. Tat pathway signal sequence domain protein [*Cutibacterium acnes* HL043PA2] | 1. 30,5829 | 1. 8,53 | 1. 293 |
| 1. 327449890 | 1. myo-inositol catabolism protein IolB [*Cutibacterium acnes* HL043PA2] | 1. 31,7904 | 1. 22,76 | 1. 290 |
| 1. 327445976 | 1. hypothetical protein HMPREF9571_01716 [*Cutibacterium acnes* HL043PA2] | 1. 30,0844 | 1. 30,34 | 1. 290 |
| 1. 327445184 | 1. oxidoreductase, aldo/keto reductase family protein [*Cutibacterium acnes* HL043PA2] | 1. 31,525 | 1. 11,85 | 1. 287 |
| 1. 327449149 | 1. tetrahydrofolate dehydrogenase/cyclohydrolase, NAD(P)-binding domain protein [*Cutibacterium acnes* HL043PA2] | 1. 30,2778 | 1. 39,37 | 1. 287 |
| 1. 327443795 | 1. hypothetical protein HMPREF9571_02536 [*Cutibacterium acnes* HL043PA2] | 1. 30,3937 | 1. 30,18 | 1. 285 |
| 1. 327444372 | 1. ATP phosphoribosyltransferase [*Cutibacterium acnes* HL043PA2] | 1. 30,5918 | 1. 15,14 | 1. 284 |
| 1. 327445930 | 1. universal stress family protein [*Cutibacterium acnes* HL043PA2] | 1. 29,2281 | 1. 15,14 | 1. 284 |
| 1. 327444215 | 1. ribosomal protein S2 [*Cutibacterium acnes* HL043PA2] | 1. 31,5164 | 1. 32,51 | 1. 283 |
| 1. 327449153 | 1. putative formyltetrahydrofolate deformylase [*Cutibacterium acnes* HL043PA2] | 1. 31,1939 | 1. 10,25 | 1. 283 |
| 1. 327445745 | 1. hypothetical protein HMPREF9571_01481 [*Cutibacterium acnes* HL043PA2] | 1. 29,8242 | 1. 6,05 | 1. 281 |
| 1. 327444299 | 1. acyl-CoA thioester hydrolase, YbgC/YbaW family [*Cutibacterium acnes* HL043PA2] | 1. 30,8817 | 1. 7,47 | 1. 281 |
| 1. 327449428 | 1. methionine aminopeptidase, type I [*Cutibacterium acnes* HL043PA2] | 1. 29,959 | 1. 7,53 | 1. 279 |
| 1. 327445997 | 1. pyridoxal 5'-phosphate synthase, synthase subunit Pdx1 [*Cutibacterium acnes* HL043PA2] | 1. 29,1149 | 1. 68,46 | 1. 279 |
| 1. 327449403 | 1. ribosomal protein L2 [*Cutibacterium acnes* HL043PA2] | 1. 30,2282 | 1. 17,63 | 1. 278 |
| 1. 327445737 | 1. proteasome, beta subunit [*Cutibacterium acnes* HL043PA2] | 1. 29,5598 | 1. 8,63 | 1. 278 |
| 1. 327446080 | 1. putative hydroxyethylthiazole kinase [*Cutibacterium acnes* HL043PA2] | 1. 28,3904 | 1. 12,64 | 1. 277 |
| 1. 327442866 | 1. NADH dehydrogenase, C subunit [*Cutibacterium acnes* HL043PA2] | 1. 30,569 | 1. 11,68 | 1. 274 |
| 1. 327445939 | 1. diaminopimelate epimerase [*Cutibacterium acnes* HL043PA2] | 1. 29,7795 | 1. 8,79 | 1. 273 |
| 1. 327447715 | 1. oxidoreductase, aldo/keto reductase family protein [*Cutibacterium acnes* HL043PA2] | 1. 29,721 | 1. 12,09 | 1. 273 |
| 1. 327445719 | 1. glycosyltransferase, group 2 family protein [*Cutibacterium acnes* HL043PA2] | 1. 30,1724 | 1. 10,29 | 1. 272 |
| 1. 327443369 | 1. putative HAD hydrolase, TIGR01457 family [*Cutibacterium acnes* HL043PA2] | 1. 29,732 | 1. 12,87 | 1. 272 |
| 1. 327449116 | 1. hypothetical protein HMPREF9571_00834 [*Cutibacterium acnes* HL043PA2] | 1. 29,7289 | 1. 20,59 | 1. 272 |
| 1. 327451135 | 1. hypothetical protein HMPREF9571_00078 [*Cutibacterium acnes* HL043PA2] | 1. 29,1008 | 1. 15,13 | 1. 271 |
| 1. 327444158 | 1. hypothetical protein HMPREF9571_02289 [*Cutibacterium acnes* HL043PA2] | 1. 29,2495 | 1. 8,86 | 1. 271 |
| 1. 327444214 | 1. translation elongation factor Ts [*Cutibacterium acnes* HL043PA2] | 1. 28,4705 | 1. 31,85 | 1. 270 |
| 1. 327449173 | 1. putative purine nucleotide phosphorylase [*Cutibacterium acnes* HL043PA2] | 1. 27,6565 | 1. 30 | 1. 270 |
| 1. 327450249 | 1. DoxX family protein [*Cutibacterium acnes* HL043PA2] | 1. 29,4094 | 1. 14,81 | 1. 270 |
| 1. 327449406 | 1. ribosomal protein S3 [*Cutibacterium acnes* HL043PA2] | 1. 29,6556 | 1. 34,57 | 1. 269 |
| 1. 327449069 | 1. aminotransferase, class IV [*Cutibacterium acnes* HL043PA2] | 1. 28,8386 | 1. 11,52 | 1. 269 |
| 1. 327446535 | 1. CobQ/CobB/MinD/ParA nucleotide binding domain protein, partial [*Cutibacterium acnes* HL043PA2] | 1. 27,8867 | 1. 7,84 | 1. 268 |
| 1. 327445028 | 1. PspA/IM30 family protein [*Cutibacterium acnes* HL043PA2] | 1. 29,5078 | 1. 46,44 | 1. 267 |
| 1. 327449184 | 1. putative phosphomethylpyrimidine kinase [*Cutibacterium acnes* HL043PA2] | 1. 27,9133 | 1. 23,22 | 1. 267 |
| 1. 327446560 | 1. oxidoreductase, short chain dehydrogenase/reductase family protein [*Cutibacterium acnes* HL043PA2] | 1. 28,5475 | 1. 38,35 | 1. 266 |
| 1. 327445789 | 1. DNA-binding regulatory protein, YebC/PmpR family [*Cutibacterium acnes* HL043PA2] | 1. 28,7785 | 1. 7,58 | 1. 264 |
| 1. 327449745 | 1. oxidoreductase, short chain dehydrogenase/reductase family protein [*Cutibacterium acnes* HL043PA2] | 1. 28,1674 | 1. 16,67 | 1. 264 |
| 1. 327445696 | 1. ATP synthase F1, delta subunit [*Cutibacterium acnes* HL043PA2] | 1. 28,4299 | 1. 26,62 | 1. 263 |
| 1. 327442868 | 1. NADH dehydrogenase subunit E [*Cutibacterium acnes* HL043PA2] | 1. 28,4199 | 1. 19,92 | 1. 261 |
| 1. 327444226 | 1. ABC transporter, ATP-binding protein [*Cutibacterium acnes* HL043PA2] | 1. 28,0096 | 1. 17,24 | 1. 261 |
| 1. 327446142 | 1. triose-phosphate isomerase [*Cutibacterium acnes* HL043PA2] | 1. 27,4409 | 1. 22,01 | 1. 259 |
| 1. 327449756 | 1. phosphate ABC transporter, ATP-binding protein [*Cutibacterium acnes* HL043PA2] | 1. 28,3666 | 1. 8,14 | 1. 258 |
| 1. 327449863 | 1. uroporphyrinogen-III C-methyltransferase [*Cutibacterium acnes* HL043PA2] | 1. 26,7818 | 1. 31,78 | 1. 258 |
| 1. 327444242 | 1. FeS assembly ATPase SufC [*Cutibacterium acnes* HL043PA2] | 1. 27,9633 | 1. 43,97 | 1. 257 |
| 1. 327444112 | 1. succinate dehydrogenase/fumarate reductase iron-sulfur subunit [*Cutibacterium acnes* HL043PA2] | 1. 27,5455 | 1. 14,4 | 1. 257 |
| 1. 327444228 | 1. putative enoyl-(acyl carrier protein) reductase [*Cutibacterium acnes* HL043PA2] | 1. 27,214 | 1. 13,62 | 1. 257 |
| 1. 327445081 | 1. putative zinc ribbon domain protein [*Cutibacterium acnes* HL043PA2] | 1. 28,6266 | 1. 9,38 | 1. 256 |
| 1. 327449702 | 1. LamB/YcsF family protein [*Cutibacterium acnes* HL043PA2] | 1. 26,5104 | 1. 15,63 | 1. 256 |
| 1. 327449227 | 1. tRNA nucleotidyltransferase [*Cutibacterium acnes* HL043PA2] | 1. 27,1039 | 1. 14,17 | 1. 254 |
| 1. 327449867 | 1. hypothetical protein HMPREF9571_00433 [*Cutibacterium acnes* HL043PA2] | 1. 28,3866 | 1. 10,24 | 1. 254 |
| 1. 327449784 | 1. phosphoglycerate mutase 1 family [*Cutibacterium acnes* HL043PA2] | 1. 27,9732 | 1. 34,54 | 1. 249 |
| 1. 327446150 | 1. phosphoglycerate mutase family protein [*Cutibacterium acnes* HL043PA2] | 1. 26,9457 | 1. 7,66 | 1. 248 |
| 1. 327444159 | 1. dihydrodipicolinate reductase [*Cutibacterium acnes* HL043PA2] | 1. 25,928 | 1. 13,82 | 1. 246 |
| 1. 327443872 | 1. pseudouridylate synthase [*Cutibacterium acnes* HL043PA2] | 1. 27,3936 | 1. 16,33 | 1. 245 |
| 1. 327449769 | 1. oxidoreductase, short chain dehydrogenase/reductase family protein [*Cutibacterium acnes* HL043PA2] | 1. 25,701 | 1. 14,69 | 1. 245 |
| 1. 327445900 | 1. hypothetical protein HMPREF9571_01640 [*Cutibacterium acnes* HL043PA2] | 1. 26,91 | 1. 46,72 | 1. 244 |
| 1. 327447632 | 1. response regulator receiver domain protein [*Cutibacterium acnes* HL043PA2] | 1. 27,3162 | 1. 13,58 | 1. 243 |
| 1. 327450482 | 1. cyclic nucleotide-binding domain protein [*Cutibacterium acnes* HL043PA2] | 1. 26,2065 | 1. 11,93 | 1. 243 |
| 1. 327449720 | 1. uroporphyrinogen-III synthase [*Cutibacterium acnes* HL043PA2] | 1. 25,1626 | 1. 10,37 | 1. 241 |
| 1. 327449923 | 1. iron dependent repressor DNA binding domain protein [*Cutibacterium acnes* HL043PA2] | 1. 26,2862 | 1. 10 | 1. 240 |
| 1. 327445326 | 1. FCD domain protein [*Cutibacterium acnes* HL043PA2] | 1. 26,0642 | 1. 20,08 | 1. 239 |
| 1. 327443896 | 1. transcriptional regulator, IclR family, C-terminal domain protein [*Cutibacterium acnes* HL043PA2] | 1. 24,9494 | 1. 14,64 | 1. 239 |
| 1. 327444201 | 1. TENA/THI-4 family protein [*Cutibacterium acnes* HL043PA2] | 1. 26,6043 | 1. 10,46 | 1. 239 |
| 1. 327444213 | 1. UMP kinase [*Cutibacterium acnes* HL043PA2] | 1. 25,6977 | 1. 36,13 | 1. 238 |
| 1. 327445958 | 1. orotidine 5'-phosphate decarboxylase [*Cutibacterium acnes* HL043PA2] | 1. 24,3475 | 1. 16,53 | 1. 236 |
| 1. 327446551 | 1. glycosyltransferase, group 1 family protein [*Cutibacterium acnes* HL043PA2] | 1. 25,1218 | 1. 11,54 | 1. 234 |
| 1. 327445671 | 1. RNA polymerase sigma-70 factor [*Cutibacterium acnes* HL043PA2] | 1. 26,3122 | 1. 11,11 | 1. 234 |
| 1. 327446960 | 1. ribosomal protein L1 [*Cutibacterium acnes* HL043PA2] | 1. 24,7229 | 1. 43,1 | 1. 232 |
| 1. 327445157 | 1. DJ-1/PfpI family protein [*Cutibacterium acnes* HL043PA2] | 1. 24,4872 | 1. 10,92 | 1. 229 |
| 1. 327448381 | 1. putative N-acetylmannosamine-6-phosphate epimerase [*Cutibacterium acnes* HL043PA2] | 1. 24,0112 | 1. 31,44 | 1. 229 |
| 1. 327449423 | 1. glycerophosphodiester phosphodiesterase family protein [*Cutibacterium acnes* HL043PA2] | 1. 25,1787 | 1. 13,16 | 1. 228 |
| 1. 327443909 | 1. cell division ATP-binding protein FtsE [*Cutibacterium acnes* HL043PA2] | 1. 25,5515 | 1. 8,77 | 1. 228 |
| 1. 327446163 | 1. periplasmic binding protein [*Cutibacterium acnes* HL043PA2] | 1. 24,6392 | 1. 12,78 | 1. 227 |
| 1. 327445736 | 1. proteasome, alpha subunit [*Cutibacterium acnes* HL043PA2] | 1. 24,2974 | 1. 7,96 | 1. 226 |
| 1. 327450420 | 1. hypothetical protein HMPREF9571_00142 [*Cutibacterium acnes* HL043PA2] | 1. 23,9674 | 1. 7,08 | 1. 226 |
| 1. 327449779 | 1. response regulator receiver domain protein [*Cutibacterium acnes* HL043PA2] | 1. 25,1562 | 1. 12,83 | 1. 226 |
| 1. 327445897 | 1. TrkA N-terminal domain protein [*Cutibacterium acnes* HL043PA2] | 1. 24,734 | 1. 22,22 | 1. 225 |
| 1. 327449400 | 1. 50S ribosomal protein L3 [*Cutibacterium acnes* HL043PA2] | 1. 23,4984 | 1. 28,25 | 1. 223 |
| 1. 327446018 | 1. response regulator receiver domain protein [*Cutibacterium acnes* HL043PA2] | 1. 23,9958 | 1. 8,07 | 1. 223 |
| 1. 327445744 | 1. ribulose-phosphate 3-epimerase [*Cutibacterium acnes* HL043PA2] | 1. 24,1126 | 1. 16,14 | 1. 223 |
| 1. 327449781 | 1. phosphate transport system regulatory protein PhoU [*Cutibacterium acnes* HL043PA2] | 1. 24,4103 | 1. 18,47 | 1. 222 |
| 1. 327448425 | 1. co-chaperone GrpE [*Cutibacterium acnes* HL043PA2] | 1. 24,3015 | 1. 12,22 | 1. 221 |
| 1. 327445702 | 1. ATP:cob(I)alamin adenosyltransferase [*Cutibacterium acnes* HL043PA2] | 1. 24,0792 | 1. 15,84 | 1. 221 |
| 1. 327445908 | 1. hypothetical protein HMPREF9571_01648 [*Cutibacterium acnes* HL043PA2] | 1. 23,7866 | 1. 12,27 | 1. 220 |
| 1. 327445296 | 1. hypothetical protein HMPREF9571_02000 [*Cutibacterium acnes* HL043PA2] | 1. 23,1003 | 1. 26,15 | 1. 218 |
| 1. 327443376 | 1. dihydroxyacetone kinase, L subunit [*Cutibacterium acnes* HL043PA2] | 1. 22,1231 | 1. 32,57 | 1. 218 |
| 1. 327445092 | 1. O-methyltransferase [*Cutibacterium acnes* HL043PA2] | 1. 23,3181 | 1. 11,06 | 1. 217 |
| 1. 327445957 | 1. orotate phosphoribosyltransferase [*Cutibacterium acnes* HL043PA2] | 1. 23,0188 | 1. 13,89 | 1. 216 |
| 1. 327446957 | 1. preprotein translocase, SecE subunit [*Cutibacterium acnes* HL043PA2] | 1. 23,5371 | 1. 16,2 | 1. 216 |
| 1. 327446187 | 1. response regulator receiver domain protein [*Cutibacterium acnes* HL043PA2] | 1. 23,8211 | 1. 37,67 | 1. 215 |
| 1. 327449070 | 1. transcriptional regulator, TetR family [*Cutibacterium acnes* HL043PA2] | 1. 23,3531 | 1. 26,05 | 1. 215 |
| 1. 327445339 | 1. hypothetical protein HMPREF9571_01977 [*Cutibacterium acnes* HL043PA2] | 1. 23,2977 | 1. 8,37 | 1. 215 |
| 1. 327449417 | 1. ribosomal protein S5 [*Cutibacterium acnes* HL043PA2] | 1. 22,5519 | 1. 30,7 | 1. 215 |
| 1. 327444369 | 1. translation initiation factor IF-3 [*Cutibacterium acnes* HL043PA2] | 1. 24,0418 | 1. 27,23 | 1. 213 |
| 1. 327443802 | 1. ribosomal subunit interface protein [*Cutibacterium acnes* HL043PA2] | 1. 23,7463 | 1. 37,74 | 1. 212 |
| 1. 327445822 | 1. TIGR03085 family protein [*Cutibacterium acnes* HL043PA2] | 1. 23,1272 | 1. 13,74 | 1. 211 |
| 1. 327449412 | 1. ribosomal protein L5 [*Cutibacterium acnes* HL043PA2] | 1. 23,8435 | 1. 35,24 | 1. 210 |
| 1. 327445670 | 1. DoxX family protein [*Cutibacterium acnes* HL043PA2] | 1. 21,9569 | 1. 29,52 | 1. 210 |
| 1. 327451126 | 1. uracil phosphoribosyltransferase [*Cutibacterium acnes* HL043PA2] | 1. 22,6387 | 1. 17,79 | 1. 208 |
| 1. 327451297 | 1. hypothetical protein HMPREF9571_00027 [*Cutibacterium acnes* HL043PA2] | 1. 21,9161 | 1. 10,14 | 1. 207 |
| 1. 327449144 | 1. phosphoribosylglycinamide formyltransferase [*Cutibacterium acnes* HL043PA2] | 1. 22,2486 | 1. 11,11 | 1. 207 |
| 1. 327449980 | 1. ribosomal protein L25, Ctc-form [*Cutibacterium acnes* HL043PA2] | 1. 22,3085 | 1. 7,8 | 1. 205 |
| 1. 327446566 | 1. bacterial transferase hexapeptide repeat protein [*Cutibacterium acnes* HL043PA2] | 1. 21,7039 | 1. 31,71 | 1. 205 |
| 1. 327449442 | 1. superoxide dismutase [*Cutibacterium acnes* HL043PA2] | 1. 23,3234 | 1. 8,29 | 1. 205 |
| 1. 327446961 | 1. ribosomal protein L10 [*Cutibacterium acnes* HL043PA2] | 1. 20,9161 | 1. 50,49 | 1. 204 |
| 1. 327449141 | 1. riboflavin synthase, alpha subunit [*Cutibacterium acnes* HL043PA2] | 1. 21,5371 | 1. 15,69 | 1. 204 |
| 1. 327446728 | 1. R3H domain protein [*Cutibacterium acnes* HL043PA2] | 1. 22,2115 | 1. 8,87 | 1. 203 |
| 1. 327444311 | 1. oligoribonuclease [*Cutibacterium acnes* HL043PA2] | 1. 22,5422 | 1. 23,27 | 1. 202 |
| 1. 327449433 | 1. ribosomal protein S4 [*Cutibacterium acnes* HL043PA2] | 1. 23,1714 | 1. 35,82 | 1. 201 |
| 1. 327442872 | 1. NADH-quinone oxidoreductase, chain I [*Cutibacterium acnes* HL043PA2] | 1. 22,3223 | 1. 17,17 | 1. 198 |
| 1. 327445981 | 1. pyridoxal phosphate enzyme, YggS family [*Cutibacterium acnes* HL043PA2] | 1. 21,785 | 1. 11,11 | 1. 198 |
| 1. 327445869 | 1. FHA domain protein [*Cutibacterium acnes* HL043PA2] | 1. 20,4459 | 1. 22,4 | 1. 192 |
| 1. 327450463 | 1. transcriptional regulator, PadR family [*Cutibacterium acnes* HL043PA2] | 1. 21,441 | 1. 13,61 | 1. 191 |
| 1. 327445965 | 1. pyrimidine operon regulatory protein/uracil phosphoribosyltransferase PyrR [*Cutibacterium acnes* HL043PA2] | 1. 20,8941 | 1. 46,6 | 1. 191 |
| 1. 327445757 | 1. translation elongation factor P [*Cutibacterium acnes* HL043PA2] | 1. 21,1367 | 1. 8,47 | 1. 189 |
| 1. 327449427 | 1. adenylate kinase [*Cutibacterium acnes* HL043PA2] | 1. 20,8857 | 1. 35,45 | 1. 189 |
| 1. 327448439 | 1. peroxiredoxin [*Cutibacterium acnes* HL043PA2] | 1. 20,8042 | 1. 35,29 | 1. 187 |
| 1. 327444212 | 1. ribosome recycling factor [*Cutibacterium acnes* HL043PA2] | 1. 20,9168 | 1. 38,04 | 1. 184 |
| 1. 327450262 | 1. inorganic diphosphatase [*Cutibacterium acnes* HL043PA2] | 1. 20,7478 | 1. 10,33 | 1. 184 |
| 1. 327445695 | 1. ATP synthase F0, B subunit [*Cutibacterium acnes* HL043PA2] | 1. 20,1494 | 1. 11,96 | 1. 184 |
| 1. 327449105 | 1. single-strand binding family protein [*Cutibacterium acnes* HL043PA2] | 1. 19,3752 | 1. 16,85 | 1. 184 |
| 1. 327449415 | 1. ribosomal protein L6 [*Cutibacterium acnes* HL043PA2] | 1. 19,8247 | 1. 31,67 | 1. 180 |
| 1. 327449435 | 1. ribosomal protein L17 [*Cutibacterium acnes* HL043PA2] | 1. 19,1911 | 1. 20,11 | 1. 179 |
| 1. 327444116 | 1. 16S rRNA processing protein RimM [*Cutibacterium acnes* HL043PA2] | 1. 19,428 | 1. 13,97 | 1. 179 |
| 1. 327451159 | 1. intracellular protease, PfpI family [*Cutibacterium acnes* HL043PA2] | 1. 19,0128 | 1. 16,76 | 1. 179 |
| 1. 327444189 | 1. hypothetical protein HMPREF9571_02320 [*Cutibacterium acnes* HL043PA2] | 1. 19,5229 | 1. 9,55 | 1. 178 |
| 1. 327450229 | 1. hypothetical protein HMPREF9571_00209 [*Cutibacterium acnes* HL043PA2] | 1. 18,1967 | 1. 19,1 | 1. 178 |
| 1. 327445782 | 1. adenine phosphoribosyltransferase [*Cutibacterium acnes* HL043PA2] | 1. 18,3547 | 1. 20,9 | 1. 177 |
| 1. 327447720 | 1. ferritin-like protein [*Cutibacterium acnes* HL043PA2] | 1. 19,0296 | 1. 18,18 | 1. 176 |
| 1. 327450278 | 1. 2-amino-4-hydroxy-6-hydroxymethyldihydropteridine diphosphokinase [*Cutibacterium acnes* HL043PA2] | 1. 19,0767 | 1. 16,57 | 1. 175 |
| 1. 327448385 | 1. hypothetical protein HMPREF9571_00868 [*Cutibacterium acnes* HL043PA2] | 1. 19,1431 | 1. 13,14 | 1. 175 |
| 1. 327446588 | 1. hypothetical protein HMPREF9571_01154 [*Cutibacterium acnes* HL043PA2] | 1. 19,9241 | 1. 20,69 | 1. 174 |
| 1. 327446831 | 1. hypothetical protein HMPREF9571_01086 [*Cutibacterium acnes* HL043PA2] | 1. 19,7591 | 1. 9,77 | 1. 174 |
| 1. 327449457 | 1. ribosomal protein S9 [*Cutibacterium acnes* HL043PA2] | 1. 18,5878 | 1. 20,81 | 1. 173 |
| 1. 327446196 | 1. hypothetical protein HMPREF9571_01940 [*Cutibacterium acnes* HL043PA2] | 1. 19,4377 | 1. 13,95 | 1. 172 |
| 1. 327449110 | 1. ferritin-like protein [*Cutibacterium acnes* HL043PA2] | 1. 19,0304 | 1. 36,05 | 1. 172 |
| 1. 327449808 | 1. hypothetical protein HMPREF9571_00374 [*Cutibacterium acnes* HL043PA2] | 1. 18,5022 | 1. 18,02 | 1. 172 |
| 1. 327445149 | 1. ACT domain protein [*Cutibacterium acnes* HL043PA2] | 1. 18,3715 | 1. 13,95 | 1. 172 |
| 1. 327449988 | 1. hypothetical protein HMPREF9571_00558 [*Cutibacterium acnes* HL043PA2] | 1. 17,9651 | 1. 11,7 | 1. 171 |
| 1. 327445350 | 1. peptidyl-prolyl cis-trans isomerase, cyclophilin-type [*Cutibacterium acnes* HL043PA2] | 1. 18,3522 | 1. 22,35 | 1. 170 |
| 1. 327445178 | 1. prokaryotic transcription elongation factor, GreA/GreB domain protein [*Cutibacterium acnes* HL043PA2] | 1. 18,3008 | 1. 12,5 | 1. 168 |
| 1. 327444240 | 1. SUF system FeS assembly protein, NifU family [*Cutibacterium acnes* HL043PA2] | 1. 18,2898 | 1. 22,89 | 1. 166 |
| 1. 327449143 | 1. 6,7-dimethyl-8-ribityllumazine synthase [*Cutibacterium acnes* HL043PA2] | 1. 16,7725 | 1. 10,84 | 1. 166 |
| 1. 327445050 | 1. PTS system, glucose subfamily, IIA component [*Cutibacterium acnes* HL043PA2] | 1. 16,8165 | 1. 15,24 | 1. 164 |
| 1. 327449777 | 1. CarD-like protein [*Cutibacterium acnes* HL043PA2] | 1. 18,1987 | 1. 30,43 | 1. 161 |
| 1. 327446193 | 1. C4-type zinc finger protein, DksA/TraR family [*Cutibacterium acnes* HL043PA2] | 1. 17,2182 | 1. 9,43 | 1. 159 |
| 1. 327451171 | 1. hypothetical protein HMPREF9571_00115, partial [*Cutibacterium acnes* HL043PA2] | 1. 16,6993 | 1. 14,56 | 1. 158 |
| 1. 327444290 | 1. ribose 5-phosphate isomerase [*Cutibacterium acnes* HL043PA2] | 1. 17,2045 | 1. 17,2 | 1. 157 |
| 1. 327448423 | 1. transcriptional regulator, MerR family [*Cutibacterium acnes* HL043PA2] | 1. 17,249 | 1. 10,46 | 1. 153 |
| 1. 327449405 | 1. ribosomal protein L22 [*Cutibacterium acnes* HL043PA2] | 1. 16,699 | 1. 30,07 | 1. 153 |
| 1. 327449100 | 1. phosphoenolpyruvate-dependent sugar phosphotransferase system, EIIA 2 [*Cutibacterium acnes* HL043PA2] | 1. 15,9764 | 1. 17,65 | 1. 153 |
| 1. 327445848 | 1. methylmalonyl-CoA epimerase [*Cutibacterium acnes* HL043PA2] | 1. 16,7934 | 1. 25,66 | 1. 152 |
| 1. 327445329 | 1. Hsp20/alpha crystallin family protein [*Cutibacterium acnes* HL043PA2] | 1. 16,6995 | 1. 23,68 | 1. 152 |
| 1. 327445969 | 1. hypothetical protein HMPREF9571_01709 [*Cutibacterium acnes* HL043PA2] | 1. 16,426 | 1. 19,21 | 1. 151 |
| 1. 327450481 | 1. endoribonuclease L-PSP [*Cutibacterium acnes* HL043PA2] | 1. 14,9469 | 1. 23,84 | 1. 151 |
| 1. 327449107 | 1. ribosomal protein L9 [*Cutibacterium acnes* HL043PA2] | 1. 16,108 | 1. 22,15 | 1. 149 |
| 1. 327444118 | 1. ribosomal protein S16 [*Cutibacterium acnes* HL043PA2] | 1. 15,9462 | 1. 52,38 | 1. 147 |
| 1. 327445700 | 1. ATP synthase F1, epsilon subunit [*Cutibacterium acnes* HL043PA2] | 1. 16,0202 | 1. 15,07 | 1. 146 |
| 1. 327449925 | 1. thioredoxin [*Cutibacterium acnes* HL043PA2] | 1. 16,0921 | 1. 23,45 | 1. 145 |
| 1. 327446772 | 1. OsmC-like protein [*Cutibacterium acnes* HL043PA2] | 1. 15,3427 | 1. 31,03 | 1. 145 |
| 1. 327444580 | 1. ribosomal protein S7 [*Cutibacterium acnes* HL043PA2] | 1. 16,2586 | 1. 29,17 | 1. 144 |
| 1. 327444180 | 1. ribosome-binding factor A [*Cutibacterium acnes* HL043PA2] | 1. 15,592 | 1. 36,36 | 1. 143 |
| 1. 327446959 | 1. ribosomal protein L11 [*Cutibacterium acnes* HL043PA2] | 1. 15,125 | 1. 16,78 | 1. 143 |
| 1. 327445159 | 1. peroxiredoxin, Ohr subfamily [*Cutibacterium acnes* HL043PA2] | 1. 14,7333 | 1. 28,17 | 1. 142 |
| 1. 327449945 | 1. TOBE domain protein [*Cutibacterium acnes* HL043PA2] | 1. 15,3212 | 1. 20,42 | 1. 142 |
| 1. 327446137 | 1. nucleoside diphosphate kinase [*Cutibacterium acnes* HL043PA2] | 1. 15,3429 | 1. 19,57 | 1. 138 |
| 1. 327446542 | 1. hypothetical protein HMPREF9571_01107 [*Cutibacterium acnes* HL043PA2] | 1. 15,2666 | 1. 18,98 | 1. 137 |
| 1. 327446043 | 1. DoxX family protein [*Cutibacterium acnes* HL043PA2] | 1. 13,7245 | 1. 19,55 | 1. 133 |
| 1. 327446962 | 1. ribosomal protein L7/L12 [*Cutibacterium acnes* HL043PA2] | 1. 13,6932 | 1. 28,46 | 1. 130 |
| 1. 327446536 | 1. single-strand binding family protein [*Cutibacterium acnes* HL043PA2] | 1. 14,1052 | 1. 17,97 | 1. 128 |
| 1. 327449416 | 1. ribosomal protein L18 [*Cutibacterium acnes* HL043PA2] | 1. 13,6924 | 1. 19,69 | 1. 127 |
| 1. 327446705 | 1. RbsD/FucU transport family protein [*Cutibacterium acnes* HL043PA2] | 1. 13,8192 | 1. 18,9 | 1. 127 |
| 1. 327449431 | 1. 30S ribosomal protein S13 [*Cutibacterium acnes* HL043PA2] | 1. 13,9878 | 1. 25,2 | 1. 123 |
| 1. 327444367 | 1. ribosomal protein L20 [*Cutibacterium acnes* HL043PA2] | 1. 13,9514 | 1. 21,14 | 1. 123 |
| 1. 327446218 | 1. glycine cleavage system H protein [*Cutibacterium acnes* HL043PA2] | 1. 12,9532 | 1. 16,26 | 1. 123 |
| 1. 327449410 | 1. ribosomal protein L14 [*Cutibacterium acnes* HL043PA2] | 1. 13,4704 | 1. 24,59 | 1. 122 |
| 1. 327449411 | 1. ribosomal protein L24 [*Cutibacterium acnes* HL043PA2] | 1. 13,4724 | 1. 18,03 | 1. 122 |
| 1. 327448389 | 1. methylmalonyl-CoA carboxyltransferase 1.3S subunit [*Cutibacterium acnes* HL043PA2] | 1. 11,6191 | 1. 21,67 | 1. 120 |
| 1. 327447699 | 1. hypothetical protein HMPREF9571_00982 [*Cutibacterium acnes* HL043PA2] | 1. 13,3495 | 1. 27,73 | 1. 119 |
| 1. 327444351 | 1. ribosomal protein L19 [*Cutibacterium acnes* HL043PA2] | 1. 13,0711 | 1. 28,21 | 1. 117 |
| 1. 327445025 | 1. iron-sulfur cluster assembly accessory protein [*Cutibacterium acnes* HL043PA2] | 1. 12,4641 | 1. 13,68 | 1. 117 |
| 1. 327445718 | 1. hypothetical protein HMPREF9571_01453 [*Cutibacterium acnes* HL043PA2] | 1. 12,7241 | 1. 18,18 | 1. 110 |
| 1. 327450282 | 1. hypothetical protein HMPREF9571_00263 [*Cutibacterium acnes* HL043PA2] | 1. 11,7749 | 1. 20 | 1. 105 |
| 1. 327450413 | 1. hypothetical protein HMPREF9571_00135 [*Cutibacterium acnes* HL043PA2] | 1. 11,2758 | 1. 23,81 | 1. 105 |
| 1. 327449399 | 1. ribosomal protein S10 [*Cutibacterium acnes* HL043PA2] | 1. 11,6723 | 1. 26,21 | 1. 103 |
| 1. 327451377 | 1. Ribbon-helix-helix protein, CopG family [*Cutibacterium acnes* HL043PA2] | 1. 10,9405 | 1. 31,07 | 1. 103 |
| 1. 327449402 | 1. ribosomal protein L23 [*Cutibacterium acnes* HL043PA2] | 1. 11,3232 | 1. 39,22 | 1. 102 |
| 1. 327445904 | 1. hypothetical protein HMPREF9571_01644 [*Cutibacterium acnes* HL043PA2] | 1. 11,0151 | 1. 15,31 | 1. 98 |
| 1. 327449489 | 1. chaperonin GroS [*Cutibacterium acnes* HL043PA2] | 1. 10,6216 | 1. 43,88 | 1. 98 |
| 1. 327449104 | 1. ribosomal protein S6 [*Cutibacterium acnes* HL043PA2] | 1. 11,2459 | 1. 19,79 | 1. 96 |
| 1. 327449404 | 1. ribosomal protein S19 [*Cutibacterium acnes* HL043PA2] | 1. 10,5036 | 1. 50,54 | 1. 93 |
| 1. 327446843 | 1. hypothetical protein HMPREF9571_01097 [*Cutibacterium acnes* HL043PA2] | 1. 10,24 | 1. 20,65 | 1. 92 |
| 1. 327449219 | 1. DNA-binding protein HB1 [*Cutibacterium acnes* HL043PA2] | 1. 9,5822 | 1. 28,57 | 1. 91 |
| 1. 327449409 | 1. 30S ribosomal protein S17 [*Cutibacterium acnes* HL043PA2] | 1. 10,4456 | 1. 22,22 | 1. 90 |
| 1. 327446132 | 1. ribosomal protein L27 [*Cutibacterium acnes* HL043PA2] | 1. 9,51694 | 1. 52,81 | 1. 89 |
| 1. 327446066 | 1. ribosomal protein S20 [*Cutibacterium acnes* HL043PA2] | 1. 9,69442 | 1. 14,77 | 1. 88 |
| 1. 327449771 | 1. phosphocarrier, HPr family [*Cutibacterium acnes* HL043PA2] | 1. 9,00556 | 1. 26,14 | 1. 88 |
| 1. 327444162 | 1. ribosomal protein S15 [*Cutibacterium acnes* HL043PA2] | 1. 10,0734 | 1. 18,39 | 1. 87 |
| 1. 327445978 | 1. acyl carrier protein [*Cutibacterium acnes* HL043PA2] | 1. 9,0223 | 1. 30,86 | 1. 81 |
| 1. 327448390 | 1. hypothetical protein HMPREF9571_00873 [*Cutibacterium acnes* HL043PA2] | 1. 8,78664 | 1. 22,5 | 1. 80 |
| 1. 327449106 | 1. ribosomal protein S18 [*Cutibacterium acnes* HL043PA2] | 1. 8,79208 | 1. 27,85 | 1. 79 |
| 1. 327449408 | 1. ribosomal protein L29 [*Cutibacterium acnes* HL043PA2] | 1. 8,87972 | 1. 45,45 | 1. 77 |
| 1. 327450491 | 1. hypothetical protein HMPREF9571_00120, partial [*Cutibacterium acnes* HL043PA2] | 1. 8,48723 | 1. 46,75 | 1. 77 |
| 1. 327444368 | 1. ribosomal protein L35 [*Cutibacterium acnes* HL043PA2] | 1. 7,88941 | 1. 13,89 | 1. 72 |
| 1. 327444383 | 1. CsbD-like protein [*Cutibacterium acnes* HL043PA2] | 1. 7,30578 | 1. 28,17 | 1. 71 |
| 1. 327445685 | 1. ribosomal protein L31 [*Cutibacterium acnes* HL043PA2] | 1. 7,71278 | 1. 43,48 | 1. 69 |
| 1. 327445941 | 1. hypothetical protein HMPREF9571_01681 [*Cutibacterium acnes* HL043PA2] | 1. 7,16049 | 1. 84,85 | 1. 66 |
