# Supplementary material for: Composition of the Biofilm Matrix of Cutibacterium acnes Acneic Strain RT5
Source: Front Microbiol. 2019 Jun 21;10:1284. doi: 10.3389/fmicb.2019.01284 (PMC6598116; doi:10.3389/fmicb.2019.01284)
Supplement: Supplementary file 2 [file Table_2.DOCX]

Table S2. The most abundant proteins isolated from the upper and the lower phases of matrix. Proteins which were found in both phases are labeled in grey.

| **UPPER PHASE** | | | | | | | | | | | |
| --- | --- | --- | --- | --- | --- | --- | --- | --- | --- | --- | --- |
| Accession | Description | Score | Coverage | # Proteins | # Unique Peptides | | # Peptides | # PSMs | # AAs | MW [kDa] | calc. pI |
| 327449885 | chaperonin GroL [*Cutibacterium acnes* HL043PA2] | 2224,76 | 57,35 | 1 | 26 | | 26 | 84 | 544 | 56,8 | 4,74 |
| 327447713 | hypothetical protein HMPREF9571_00996 [*Cutibacterium acnes* HL043PA2] | 2042,29 | 50,71 | 1 | 19 | | 20 | 80 | 424 | 43,2 | 9,99 |
| 327449490 | chaperonin GroL [*Cutibacterium acnes* HL043PA2] | 1986,60 | 54,61 | 1 | 25 | | 25 | 68 | 531 | 56,4 | 4,87 |
| 327446144 | glyceraldehyde-3-phosphate dehydrogenase, type I [*Cutibacterium acnes* HL043PA2] - GLYCOLYSIS | 1377,39 | 69,55 | 1 | 19 | | 19 | 56 | 335 | 35,9 | 5,76 |
| 327447627 | chaperone protein DnaK [*Cutibacterium acnes* HL043PA2] | 1086,06 | 45,06 | 1 | 24 | | 24 | 51 | 617 | 66,3 | 4,78 |
| 327447637 | pyruvate, phosphate dikinase [*Cutibacterium acnes* HL043PA2] | 942,66 | 27,18 | 1 | 19 | | 19 | 37 | 883 | 96,4 | 4,97 |
| 327445141 | methylmalonyl-CoA mutase, small subunit [*Cutibacterium acnes* HL043PA2] | 840,34 | 32,70 | 1 | 17 | | 17 | 36 | 636 | 69,1 | 4,97 |
| 327449991 | phosphopyruvate hydratase [*Cutibacterium acnes* HL043PA2] | 817,59 | 40,38 | 1 | 12 | | 12 | 36 | 426 | 45,5 | 4,70 |
| 327449137 | succinate-CoA ligase, beta subunit [*Cutibacterium acnes* HL043PA2] | 739,82 | 42,46 | 1 | 15 | | 15 | 35 | 391 | 41,3 | 4,83 |
| 327446194 | DivIVA domain protein [*Cutibacterium acnes* HL043PA2] | 732,17 | 45,15 | 1 | 15 | | 15 | 33 | 361 | 40,1 | 5,25 |
| 327448392 | conserved carboxylase domain protein [*Cutibacterium acnes* HL043PA2] | 719,84 | 22,13 | 1 | 10 | | 10 | 25 | 497 | 54,7 | 5,50 |
| 327449116 | hypothetical protein HMPREF9571_00834 [*Cutibacterium acnes* HL043PA2] | 695,63 | 61,03 | 1 | 14 | | 14 | 28 | 272 | 29,7 | 5,02 |
| 327444383 | CsbD-like protein [*Cutibacterium acnes* HL043PA2] | 681,82 | 78,87 | 1 | 7 | | 7 | 31 | 71 | 7,3 | 7,24 |
| 327449219 | DNA-binding protein HB1 [*Cutibacterium acnes* HL043PA2] | 657,58 | 69,23 | 1 | 6 | | 6 | 21 | 91 | 9,6 | 9,82 |
| 327449489 | chaperonin GroS [*Cutibacterium acnes* HL043PA2] | 646,32 | 63,27 | 1 | 5 | | 5 | 27 | 98 | 10,6 | 4,92 |
| 327449152 | malate dehydrogenase [*Cutibacterium acnes* HL043PA2] | 644,87 | 39,56 | 1 | 9 | | 9 | 19 | 364 | 38,7 | 5,48 |
| 327448391 | methylmalonyl-CoA carboxyltransferase 12S subunit [*Cutibacterium acnes* HL043PA2] | 631,26 | 26,72 | 1 | 13 | | 13 | 26 | 524 | 56,4 | 5,81 |
| 327449390 | translation elongation factor Tu [*Cutibacterium acnes* HL043PA2] | 626,59 | 28,21 | 1 | 10 | | 10 | 25 | 397 | 44,1 | 5,53 |
| 327444215 | ribosomal protein S2 [*Cutibacterium acnes* HL043PA2] | 623,35 | 34,28 | 1 | 10 | | 10 | 24 | 283 | 31,5 | 6,30 |
| 327445771 | hypothetical protein HMPREF9571_01507 [*Cutibacterium acnes* HL043PA2] | 599,83 | 25,84 | 1 | 9 | | 9 | 19 | 356 | 40,1 | 5,31 |
| 327445140 | methylmalonyl-CoA mutase large subunit [*Cutibacterium acnes* HL043PA2] | 573,65 | 23,46 | 1 | 14 | | 14 | 29 | 729 | 80,1 | 5,49 |
| 327450411 | pyruvate synthase [*Cutibacterium acnes* HL043PA2] | 565,52 | 10,88 | 1 | 12 | | 12 | 23 | 1204 | 131,8 | 5,90 |
| 327445351 | hypothetical protein HMPREF9571_01989 [*Cutibacterium acnes* HL043PA2] | 558,33 | 51,75 | 1 | 14 | | 14 | 28 | 371 | 39,1 | 6,47 |
| 327445670 | DoxX family protein [*Cutibacterium acnes* HL043PA2] | 553,45 | 35,24 | 1 | 6 | | 6 | 20 | 210 | 22,0 | 10,95 |
| 327446143 | phosphoglycerate kinase [*Cutibacterium acnes* HL043PA2] | 553,33 | 40,55 | 1 | 13 | | 13 | 26 | 402 | 42,2 | 5,15 |
| 327446961 | ribosomal protein L10 [*Cutibacterium acnes* HL043PA2] | 548,71 | 53,92 | 1 | 9 | | 9 | 18 | 204 | 20,9 | 5,50 |
| 327446565 | DegT/DnrJ/EryC1/StrS aminotransferase family protein [*Cutibacterium acnes* HL043PA2] | 531,63 | 25,95 | 1 | 7 | | 7 | 19 | 370 | 39,2 | 5,97 |
| 327445697 | ATP synthase F1, alpha subunit [*Cutibacterium acnes* HL043PA2] | 527,61 | 31,38 | 1 | 13 | | 13 | 22 | 545 | 58,9 | 5,14 |
| 327445941 | hypothetical protein HMPREF9571_01681 [*Cutibacterium acnes* HL043PA2] | 515,61 | 84,85 | 1 | 8 | | 8 | 24 | 66 | 7,2 | 4,84 |
| 327449790 | glycine hydroxymethyltransferase [*Cutibacterium acnes* HL043PA2] | 505,03 | 16,70 | 1 | 7 | | 7 | 15 | 491 | 52,9 | 6,06 |
| **LOWER PHASE** | | | | | | | | | | | |
| Accession | Description | Score | Coverage | # Proteins | # Unique Peptides | # Peptides | | # PSMs | # AAs | MW [kDa] | calc. pI |
| 327449885 | chaperonin GroL [*Cutibacterium acnes* HL043PA2] | 1439,33 | 43,93 | 1 | 21 | 21 | | 95 | 544 | 56,8 | 4,74 |
| 327449490 | chaperonin GroL [*Cutibacterium acnes* HL043PA2] | 1408,30 | 59,70 | 1 | 27 | 27 | | 84 | 531 | 56,4 | 4,87 |
| 327449991 | phosphopyruvate hydratase [*Cutibacterium acnes* HL043PA2] | 741,29 | 34,98 | 1 | 10 | 10 | | 33 | 426 | 45,5 | 4,70 |
| 327447713 | hypothetical protein HMPREF9571_00996 [*Cutibacterium acnes* HL043PA2] | 695,04 | 46,23 | 1 | 16 | 17 | | 51 | 424 | 43,2 | 9,99 |
| 327446144 | glyceraldehyde-3-phosphate dehydrogenase, type I [*Cutibacterium acnes* HL043PA2] | 510,38 | 59,40 | 1 | 15 | 15 | | 39 | 335 | 35,9 | 5,76 |
| 327445141 | methylmalonyl-CoA mutase, small subunit [*Cutibacterium acnes* HL043PA2] | 489,91 | 34,28 | 1 | 18 | 18 | | 30 | 636 | 69,1 | 4,97 |
| 327450411 | pyruvate synthase [*Cutibacterium acnes* HL043PA2] | 471,07 | 11,54 | 1 | 13 | 13 | | 28 | 1204 | 131,8 | 5,90 |
| 327449152 | malate dehydrogenase [*Cutibacterium acnes* HL043PA2] | 465,89 | 30,77 | 1 | 8 | 8 | | 23 | 364 | 38,7 | 5,48 |
| 327449390 | translation elongation factor Tu [*Cutibacterium acnes* HL043PA2] | 450,74 | 37,53 | 1 | 11 | 11 | | 29 | 397 | 44,1 | 5,53 |
| 327447637 | pyruvate, phosphate dikinase [*Cutibacterium acnes* HL043PA2] | 423,55 | 24,69 | 1 | 17 | 17 | | 32 | 883 | 96,4 | 4,97 |
| 327449137 | succinate-CoA ligase, beta subunit [*Cutibacterium acnes* HL043PA2] | 392,10 | 18,41 | 1 | 8 | 8 | | 22 | 391 | 41,3 | 4,83 |
| 327449138 | succinate-CoA ligase, alpha subunit [*Cutibacterium acnes* HL043PA2] | 381,95 | 33,33 | 1 | 7 | 7 | | 16 | 297 | 30,6 | 6,14 |
| 327448391 | methylmalonyl-CoA carboxyltransferase 12S subunit [*Cutibacterium acnes* HL043PA2] | 368,78 | 26,15 | 1 | 12 | 12 | | 21 | 524 | 56,4 | 5,81 |
| 327445351 | hypothetical protein HMPREF9571_01989 [*Cutibacterium acnes* HL043PA2] | 366,22 | 35,85 | 1 | 10 | 10 | | 18 | 371 | 39,1 | 6,47 |
| 327446194 | DivIVA domain protein [*Cutibacterium acnes* HL043PA2] | 363,20 | 39,34 | 1 | 12 | 12 | | 21 | 361 | 40,1 | 5,25 |
| 327447627 | chaperone protein DnaK [*Cutibacterium acnes* HL043PA2] | 352,96 | 31,12 | 1 | 16 | 16 | | 29 | 617 | 66,3 | 4,78 |
| 327448392 | conserved carboxylase domain protein [*Cutibacterium acnes* HL043PA2] | 346,97 | 21,33 | 1 | 11 | 11 | | 21 | 497 | 54,7 | 5,50 |
| 327449489 | chaperonin GroS [*Cutibacterium acnes* HL043PA2] | 332,35 | 35,71 | 1 | 2 | 2 | | 17 | 98 | 10,6 | 4,92 |
| 327446964 | DNA-directed RNA polymerase, beta' subunit [*Cutibacterium acnes* HL043PA2] | 327,07 | 10,83 | 1 | 14 | 14 | | 23 | 1293 | 143,3 | 6,95 |
| 327449401 | 50S ribosomal protein L4 [*Cutibacterium acnes* HL043PA2] | 302,69 | 27,24 | 1 | 7 | 7 | | 13 | 301 | 32,3 | 8,73 |
| 327445697 | ATP synthase F1, alpha subunit [*Cutibacterium acnes* HL043PA2] | 288,98 | 22,02 | 1 | 9 | 9 | | 15 | 545 | 58,9 | 5,14 |
| 327445771 | hypothetical protein HMPREF9571_01507 [*Cutibacterium acnes* HL043PA2] | 287,53 | 23,03 | 1 | 8 | 8 | | 19 | 356 | 40,1 | 5,31 |
| 327446143 | phosphoglycerate kinase [*Cutibacterium acnes* HL043PA2] | 279,50 | 45,02 | 1 | 14 | 14 | | 27 | 402 | 42,2 | 5,15 |
| 327444157 | thiamine pyrophosphate enzyme, N-terminal TPP binding domain protein [*Cutibacterium acnes* HL043PA2] | 277,71 | 10,55 | 1 | 5 | 5 | | 14 | 597 | 64,1 | 6,46 |
| 327445770 | aspartate--tRNA ligase [*Cutibacterium acnes* HL043PA2] | 272,07 | 28,55 | 1 | 13 | 13 | | 27 | 606 | 66,5 | 5,22 |
| 327450410 | putative dihydroorotate dehydrogenase 2 [*Cutibacterium acnes* HL043PA2] | 268,73 | 28,13 | 1 | 8 | 8 | | 14 | 327 | 34,7 | 4,64 |
| 327444215 | ribosomal protein S2 [*Cutibacterium acnes* HL043PA2] | 268,27 | 30,04 | 1 | 7 | 7 | | 15 | 283 | 31,5 | 6,30 |
| 327449406 | ribosomal protein S3 [*Cutibacterium acnes* HL043PA2] | 265,77 | 31,97 | 1 | 7 | 7 | | 14 | 269 | 29,7 | 10,65 |
| 327446961 | ribosomal protein L10 [*Cutibacterium acnes* HL043PA2] | 256,69 | 50,00 | 1 | 8 | 8 | | 15 | 204 | 20,9 | 5,50 |
| 327444160 | guanosine pentaphosphate synthetase I/polyribonucleotide nucleotidyltransferase [*Cutibacterium acnes* HL043PA2] | 249,29 | 20,60 | 1 | 11 | 11 | | 17 | 733 | 78,9 | 4,86 |
